# MicroRNA-193b-3p represses neuroblastoma cell growth via downregulation of *Cyclin D1*, *MCL-1* and *MYCN*

**DOI:** 10.18632/oncotarget.24793

**Published:** 2018-04-06

**Authors:** Sarah Andrea Roth, Øyvind H. Hald, Steffen Fuchs, Cecilie Løkke, Ingvild Mikkola, Trond Flægstad, Johannes Schulte, Christer Einvik

**Affiliations:** ^1^ Pediatric Research Group, Department of Clinical Medicine, Faculty of Health Science, The Arctic University of Norway – UiT, Tromsø NO-9037, Norway; ^2^ Department of Pediatrics, Division of Child and Adolescent Health, UNN – University Hospital of North-Norway, Tromsø NO-9038, Norway; ^3^ Research Group of Pharmacology, Department of Pharmacy, The Arctic University of Norway – UiT, Tromsø NO-9037, Norway; ^4^ Charité – Universitätsmedizin Berlin, Corporate Member of Freie Universität Berlin, Humboldt-Universität zu Berlin, and Berlin Institute of Health, Department of Pediatric Oncology and Hematology/Bone Marrow Transplantation, Berlin 10117, Germany; ^5^ Berlin Institute of Health (BIH), Berlin 10178, Germany

**Keywords:** neuroblastoma, miRNA, tumor suppressor, mir-193b

## Abstract

Neuroblastoma is the most common diagnosed tumor in infants and the second most common extracranial tumor of childhood. The survival rate of patients with high-risk neuroblastoma is still very low despite intensive multimodal treatments. Therefore, new treatment strategies are needed. In recent years, miRNA-based anticancer therapy has received growing attention. Advances in this novel treatment strategy strongly depends on the identification of candidate miRNAs with broad-spectrum antitumor activity. Here, we identify miR-193b as a miRNA with tumor suppressive properties. We show that miR-193b is expressed at low levels in neuroblastoma cell lines and primary tumor samples. Introduction of miR-193b mimics into nine neuroblastoma cell lines with distinct genetic characteristics significantly reduces cell growth *in vitro* independent of risk factors such as p53 functionality or *MYCN* amplification. Functionally, miR-193b induces a G1 cell cycle arrest and cell death in neuroblastoma cell lines by reducing the expression of *MYCN*, *Cyclin D1* and *MCL-1*, three important oncogenes in neuroblastoma of which inhibition has shown promising results in preclinical testing. Therefore, we suggest that miR-193b may represent a new candidate for miRNA-based anticancer therapy in neuroblastoma.

## INTRODUCTION

Neuroblastoma, a neoplasm of the sympathetic nervous system, is the most common diagnosed tumor in infants and the second most common extracranial tumor of childhood [1, 2]. During the last decades, the survival of children with neuroblastoma has significantly improved. New advances and continued research on treatment strategies are contributing to increasing survival rates [2, 3]. Today, the estimated survival of children with low and intermediate-risk neuroblastoma is >98 and 90–95%, respectively. On the contrary, the survival rate of patients with high-risk neuroblastoma is still only 40–50% despite intensive multimodal treatments [2]. More than half of high-risk neuroblastoma patients experience a relapse after treatment with tumors refractory to standard chemotherapeutic agents and there are currently no salvage regimens known to be curative for these patients [2, 4]. These patients have a very poor prognosis with a ten year overall survival rate of less than 20% [5]. Thus, improvements in survival rates for children with high-risk neuroblastoma are dependent on novel treatment approaches.

MicroRNAs (miRNAs) are small (~22 nucleotides), non-coding RNA molecules regulating the expression of their target genes at the post-transcriptional level. MiRNAs have been implicated to be involved in the regulation of virtually all physiological processes including proliferation, differentiation, apoptosis, and cell survival [6, 7]. Whole-genome miRNA profiling studies revealed that miRNAs are deregulated in most human cancers, indicating that miRNAs have critical functions in carcinogenesis and tumor progression by acting as oncogenes or tumor suppressors [7–9].

In recent years, miRNAs have been reported as valuable biomarkers for diagnosis and prognosis of cancer, and miRNA-based anticancer therapy has emerged as a promising therapeutic strategy for the treatment of human cancers [10–13].

Emerging evidence has demonstrated that miRNAs play a vital role in the initiation and progression of neuroblastoma (reviewed by [14, 15]). A number of studies have demonstrated that miRNAs are differentially expressed between high-risk and low-risk neuroblastomas [16, 17], and also between *MYCN*-amplified and non-*MYCN*-amplified neuroblastomas [18–20]. We have recently demonstrated that several miRNAs have a distinct expression pattern in isogenic neuroblastoma cell lines isolated from patients at diagnosis and at relapse after intensive treatments [21]. In addition to miRNA profiling studies, several studies have focused on individual miRNAs, and performed functional *in vitro* and *in vivo* studies to analyze their specific roles in neuroblastoma. These studies identified a number of tumor suppressive and oncogenic miRNAs involved in proliferation, metastasis and differentiation of neuroblastoma cells (reviewed by [14, 15, 22, 23]).

For instance, miR-34a, which is downregulated in neuroblastoma, exhibits potent tumor suppressive functions in neuroblastoma by inducing apoptosis, cell cycle arrest and differentiation [24–29]. The miR-17-92 cluster, a direct target of N-Myc, exhibits oncogenic functions in neuroblastoma by inhibiting neuronal differentiation, increasing cell proliferation, inhibiting apoptosis, and decreasing cell adhesion (recently reviewed by [15]).

Recent studies in mice have supported the potential of miRNA replacement therapy in neuroblastoma *in vivo* [25, 26, 30–32]. For instance, nanoparticle-based targeted delivery of miR-34a into neuroblastoma tumors in a murine orthotropic xenograft model resulted in decreased tumor growth, increased apoptosis and a reduction in vascularization [26]. Treating nude mice bearing neuroblastoma xenografts with miR-542-3p-loaded nanoparticles also decreased cell proliferation and induced apoptosis *in vivo* [32]. Thus, research on miRNA-based therapy in neuroblastoma offers a chance to develop new drugs to successfully treat high-risk neuroblastoma.

To develop miRNA-based therapeutics for high-risk neuroblastoma, identification of candidate miRNAs with broad-spectrum antitumor activity is needed. In this study, we demonstrated that treatment of neuroblastoma cell lines with miR-193b mimics strongly reduces cell viability and proliferation by inducing a G1 cell cycle arrest and cell death (mainly apoptotic). Our data identified miR-193b as a candidate for miRNA-based anticancer therapy in neuroblastoma.

## RESULTS

### Low expression of miR-193b in primary neuroblastoma tumors and cell lines

MiR-193b-3p (henceforth referred to as miR-193b) has been described as a tumor suppressor in several cancers. To investigate a potential tumor suppressive role of miR-193b in neuroblastoma, we assessed miR-193b expression in 69 primary neuroblastoma tumors previously profiled for miRNA expression by RT-qPCR [33].

**Figure 1:**
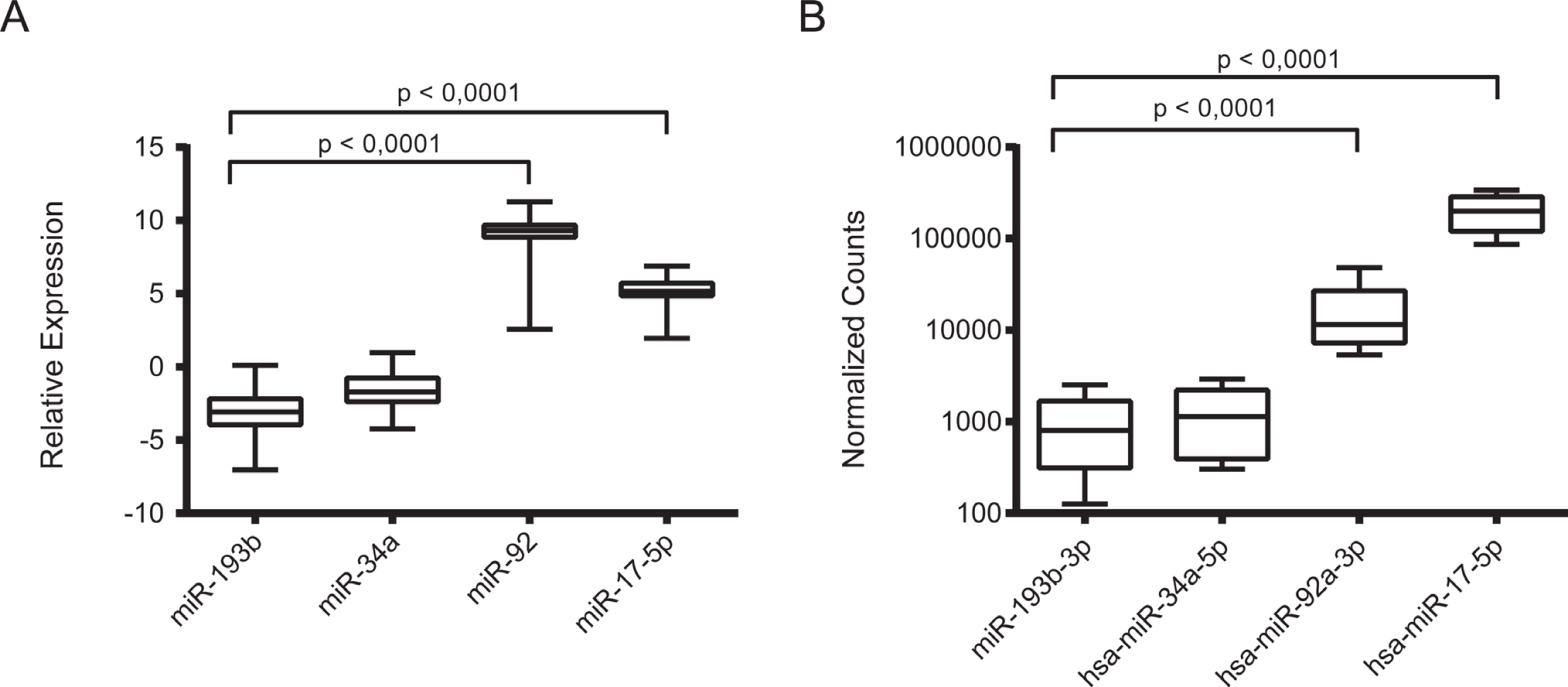
miR-193b is downregulated in primary neuroblastoma tumor samples (**A**) 69 neuroblastoma tumor samples, independent of the first cohort, were analyzed by qRT-PCR. In this cohort we also found a significant downregulation of miR-193b in comparison to the oncomiRs (*p* < 0,0001). (**B**) 10 different neuroblastoma samples were analyzed by RNA sequencing. The expression of miR-193b-3p was comparable to the expression level of the tumor suppressive miR-34a-5p and significantly lower than the expression of the known oncomiRs miR-92a-3p and miR-17-5p (*p* < 0,0001).

The expression level of miR-193b was significantly lower (*p* value < 0.0001) as compared to that of the well-defined oncogenic miRNAs miR-92a-3p and miR-17-5p (Figure 1A). In addition, the expression level of miR-193b was found to be comparable to that of miR-34a, a tumor suppressor miRNA that is expressed at low levels in unfavorable primary neuroblastoma tumors and cell lines [24]. Then, to extend the clinical data even more, we also analyzed miR-193b expression compared to miR-92a-3p and miR-17-5p expression in ten primary neuroblastoma samples by deep sequencing (Figure 1B, data from [18]). These data confirmed the RT-qPCR data indicating that miR-193b is downregulated in neuroblastoma, which points to a tumor suppressive function of miR-193b in this tumor entity. In addition, we used RT-qPCR to compare the expression of mir-193b to well established neuroblastoma oncogenic and tumor suppressor miRNAs in two neuroblastoma cell lines, Kelly and SK-N-BE(2)-C (Supplementary Figure 1). As for the tumor samples, the expression of mir-193b was significantly lower as compared to miR-92a and comparable to miR-34a in these cell lines. In concordance to these findings, analysis of miR-193b expression in neuroblastoma cell lines previously profiled by us for miRNA expression by deep sequencing [21] also revealed low expression of miR-193b when compared to known oncogenic miRNAs or tumor suppressor miRNAs, respectively (Supplementary Table 1).

### MiR-193b reduces cell viability and proliferation in neuroblastoma cell lines

**Figure 2:**
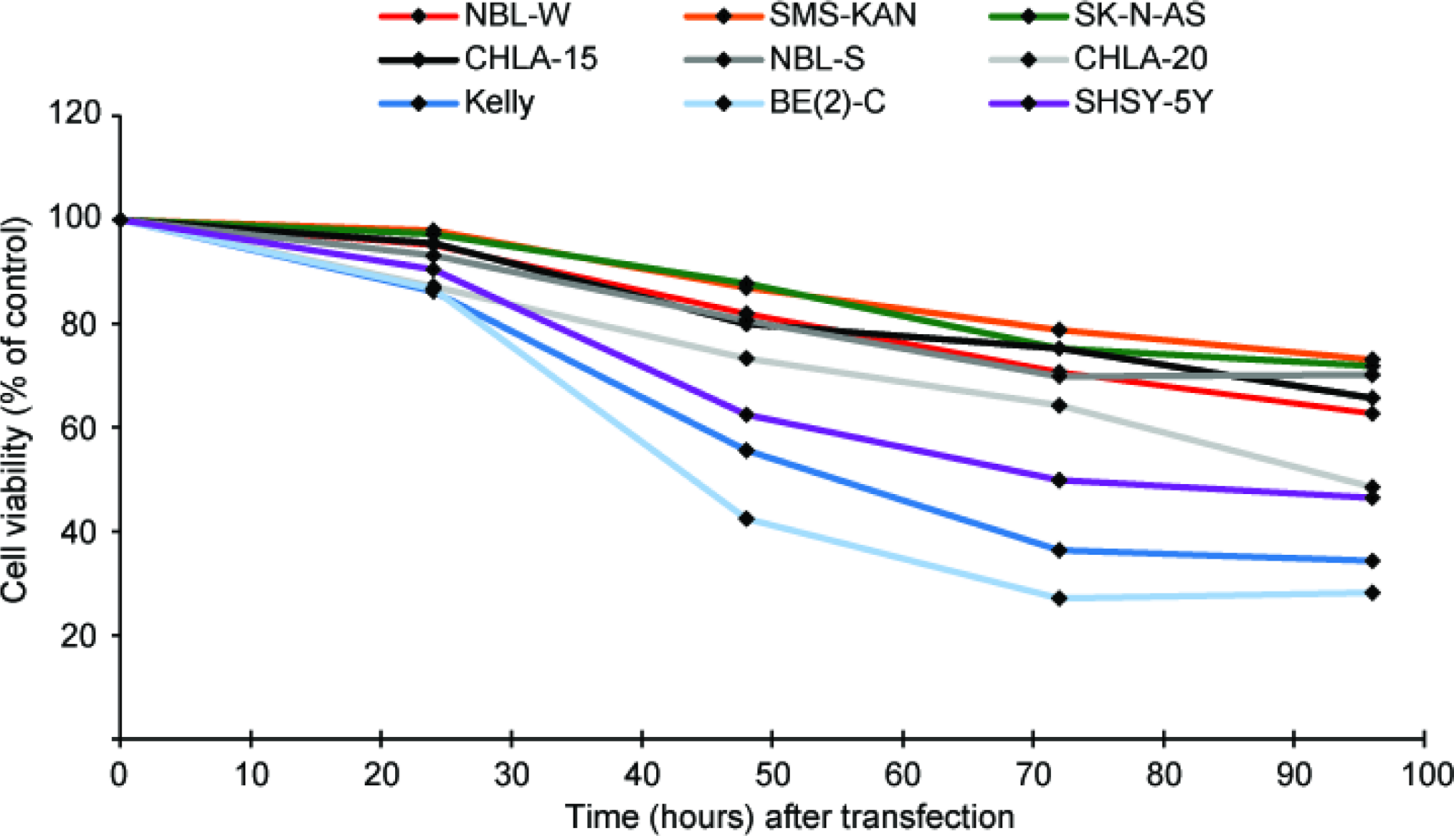
miR-193b reduces cell viability in neuroblastoma cell lines Neuroblastoma cell lines were transfected with control mimics or miR-193b mimics. Cell viability was analyzed using alamarBlue at indicated time points. The average cell viability of cells transfected with control mimics was set to 100% (not shown) and the cell viability of miR-193b-transfected cells was calculated relative to control-transfected cells. Data are mean of at least three experiments, each performed in triplicate. Standard deviations have been included in Supplementary Figure 3.

**Figure 3:**
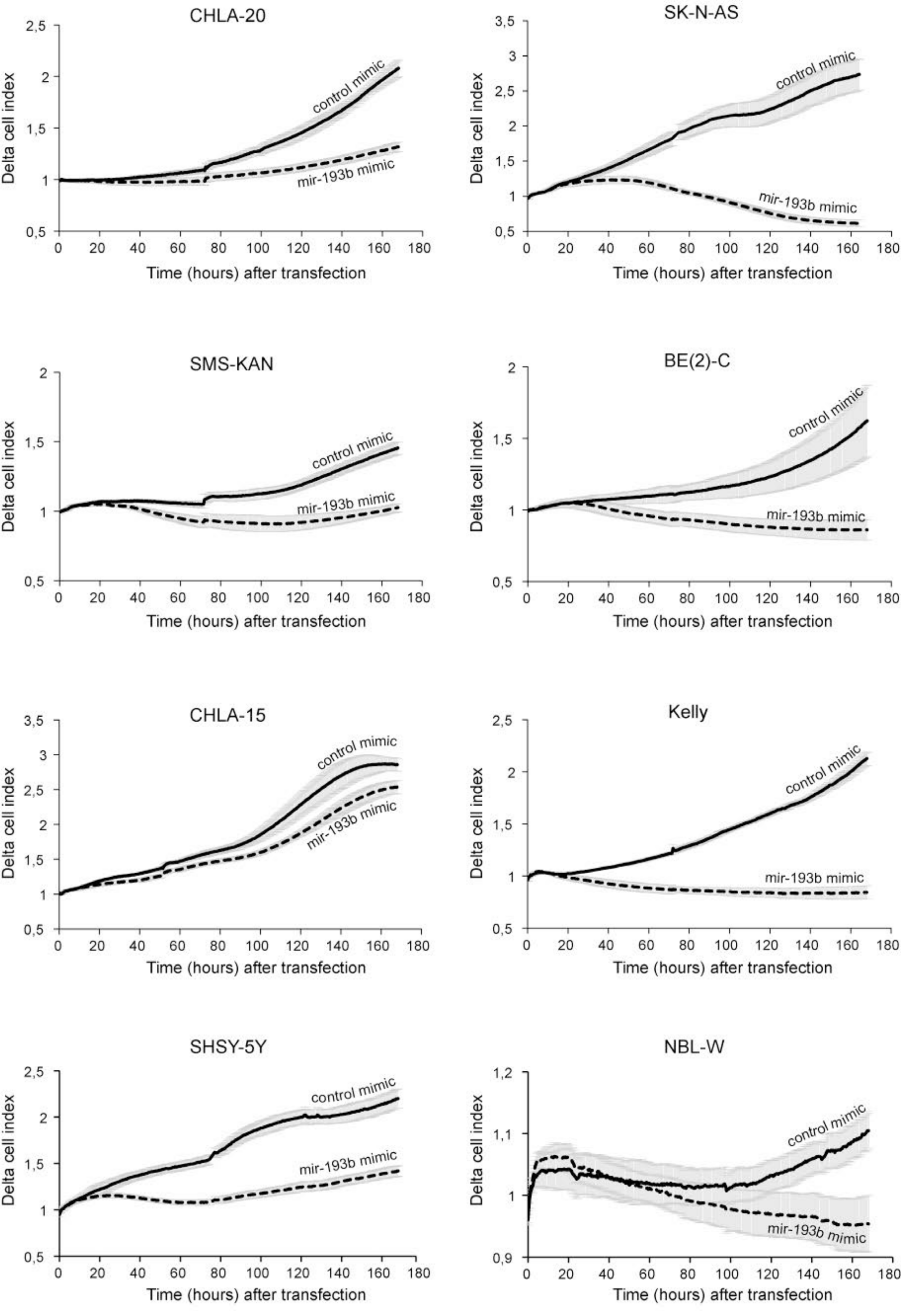
miR-193b reduces proliferation in neuroblastoma cell lines Neuroblastoma cell lines were transfected with control mimics or miR-193b mimics and cell proliferation was monitored in real time for seven days using xCELLigence. Data are mean of one representative experiment of at least two, each performed in octuplicate. (Solid line: control mimics; Dotted line: miR-193b mimics).

In order to investigate a potential tumor suppressor role of miR-193b in neuroblastoma cells, miR-193b mimics (mir-193b) or scrambled control miRNA mimics (C) were transfected into nine neuroblastoma cell lines with distinct genetic characteristics. RT-qPCR was performed to validate miR-193b overexpression (Supplementary Figure 2). As shown in Figures 2 and 3, miR-193b had a significant effect on cell viability and proliferation. In all neuroblastoma cell lines tested, a reduction in cell viability and proliferation was detected within 24 or 48 hours post-transfection, and loss of cell viability by more than 50% was apparent in SK-N-BE(2)-C (BE(2)-C), Kelly, SHSY-5Y and CHLA-20 cells 96 hours after transfection.

### MiR-193b induces caspase-dependent and -independent cell death in neuroblastoma

To determine the possible mode of the anti-proliferative activity of miR-193b, the cytotoxic effect of miR-193b was assessed following transfection of miR-193b or control mimics into each cell line. The MultiTox-Fluor Multiplex Cytotoxicity Assay simultaneously measures two protease activities; one is a marker of viability, and the other is a marker of cytotoxicity. As the ratio of viable cells to dead cells is independent of the cell number, the data can be normalized.

**Figure 4:**
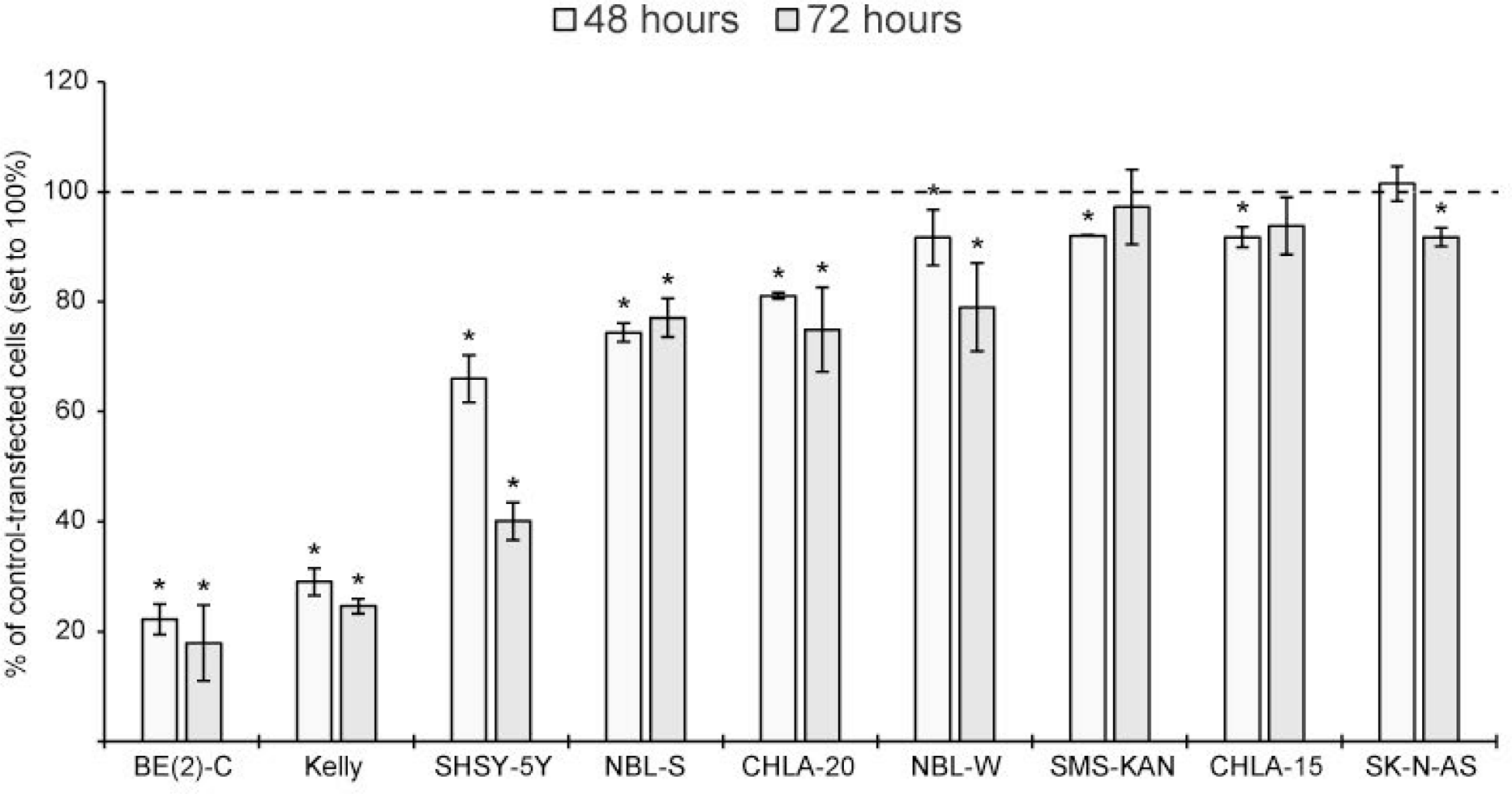
miR-193b is cytotoxic to neuroblastoma cell lines Cells were transfected with control mimics or miR-193b mimics. Cytotoxicity was analyzed at the indicated time points using the MultiToxFluor Multiplex Cytotoxicity Assay. Cytotoxicity (defined as the ratio of viable cells to dead cells) in miR-193b-transfected cells was calculated in relation to that in control-transfected cells. Data are mean ± SD of at least two independent experiments, each performed in triplicates (^*^p < 0.01 Student`s *t*-test).

High cytotoxic effects were detected in BE(2)-C, Kelly and SHSY-5Y cells by day two. Significant, but lower cytotoxic effects were observed in NBL-S, CHLA-20 and NBL-W cells. MiR-193b had only a modest cytotoxic effect (less than 10%) in SMS-KAN, CHLA-15 and SK-N-AS cells (Figure 4 ).

**Figure 5:**
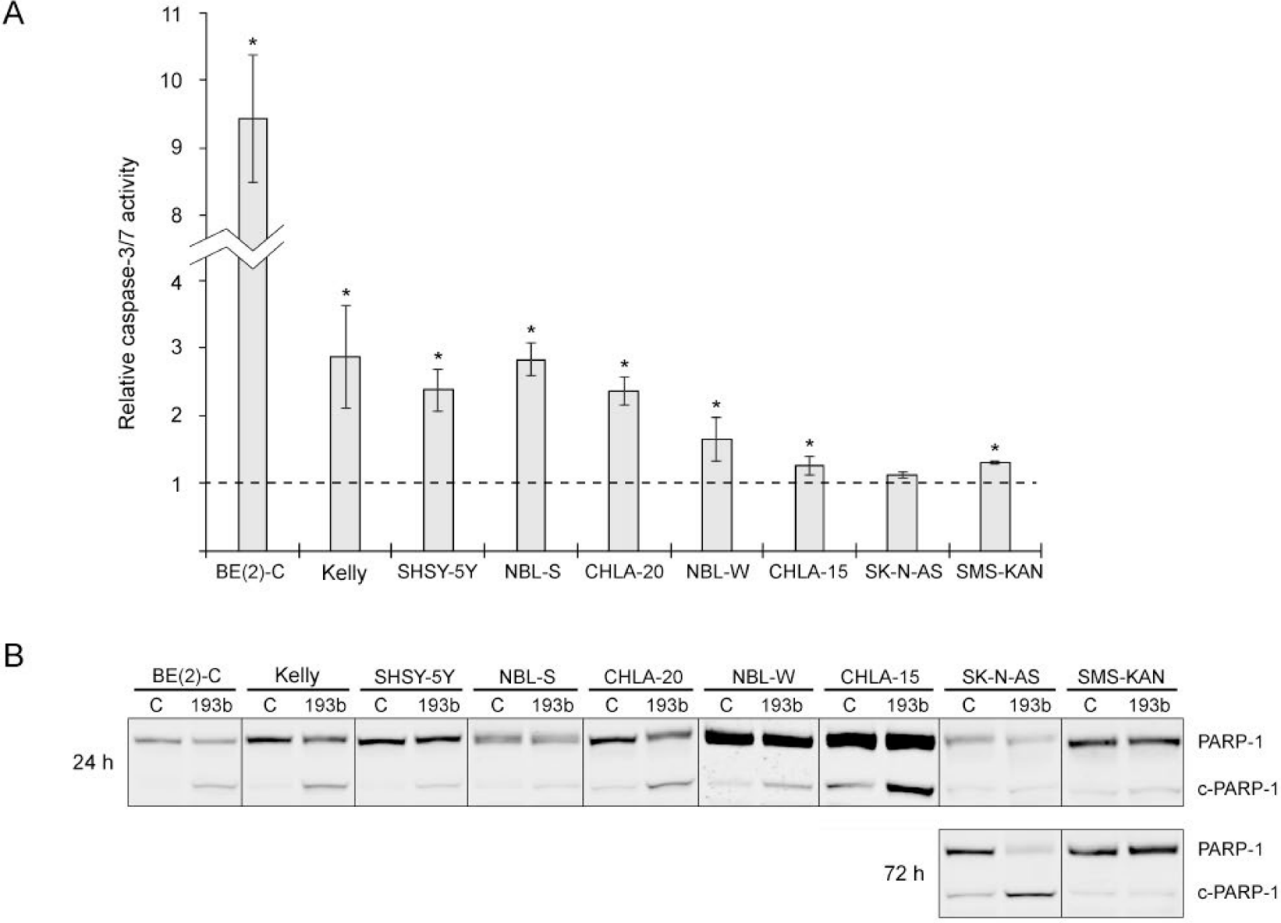
miR-193b induces apoptosis in neuroblastoma cell lines (**A**) Cells were transfected with control mimics or miR-193b mimics and caspase activity was measured 48 hours after transfection. The results are expressed as relative caspase activities of miR-193b-transfected cells, which is the ratio between the caspase activity of miR-193b-transfected cells and that measured in control-transfected cells. Data are mean ± SD of at least two independent experiment, each performed in triplicates (^*^p < 0.01 Student`s *t*-test). (**B**) Cells were transfected as in (A). Total PARP1 and PARP-1 cleavage (c-PARP-1) was assessed by Western blot 24 and 72 hours post-transfection, respectively. The experiment was performed at least two times giving similar results, and the result from one representative experiment is shown.

Next, to investigate whether the cytotoxic effect of miR-193b is mediated through the induction of apoptosis, activation of caspase-3/7 and PARP-1 cleavage was assessed following transfection of miR-193b into each cell line (Figure 5 ). In CHLA-15, SMS-KAN, SK-N-AS cells (1.12–1.31-fold relative change) and in NBL-W cells (average fold change 1.65) there was only a small increase in the caspase-3/7 enzymatic activity in miR-193b-transfected cells as compared to control-transfected cells. In BE(2)-C, SHSY-5Y, NBL-S, CHLA-20 and Kelly cells, the caspase-3/7 activity had increased more than two-fold 48 hours post-transfection (Figure 5A). In concordance with this, miR-193b mimic transfection also caused an increase in PARP-1 cleavage in BE(2)-C, Kelly, SHSY-5Y, NBL-S, CHLA-20 and NBL-W cells with a concomitant decrease in total PARP-1, whereas there was only a modest increase in PARP-1 cleavage in SK-N-AS and SMS-KAN cells (Figure 5B). To analyze whether miR-193b may induce secondary cell death, we also analyzed PARP-1 cleavage at 72 hours post-transfection in those cell lines where no PARP-1 cleavage was detected 24 hours post-transfection. 72 hours post-transfection, PARP-1 cleavage had increased in SK-N-AS but not in SMS-KAN cells (Figure 5B). Surprisingly, while miR-193b only slightly increased caspase-3/7 activity in CHLA-15 cells (average fold change 1.26), miR-193b substantially increased PARP-1 cleavage 24 hours post-transfection, which may indicate caspase-independent cell death in this cell line (Figure 5B). To summarize, these results indicate that miR-193b induces both caspase-dependent and -independent cell death in neuroblastoma cell lines.

**Figure 6:**
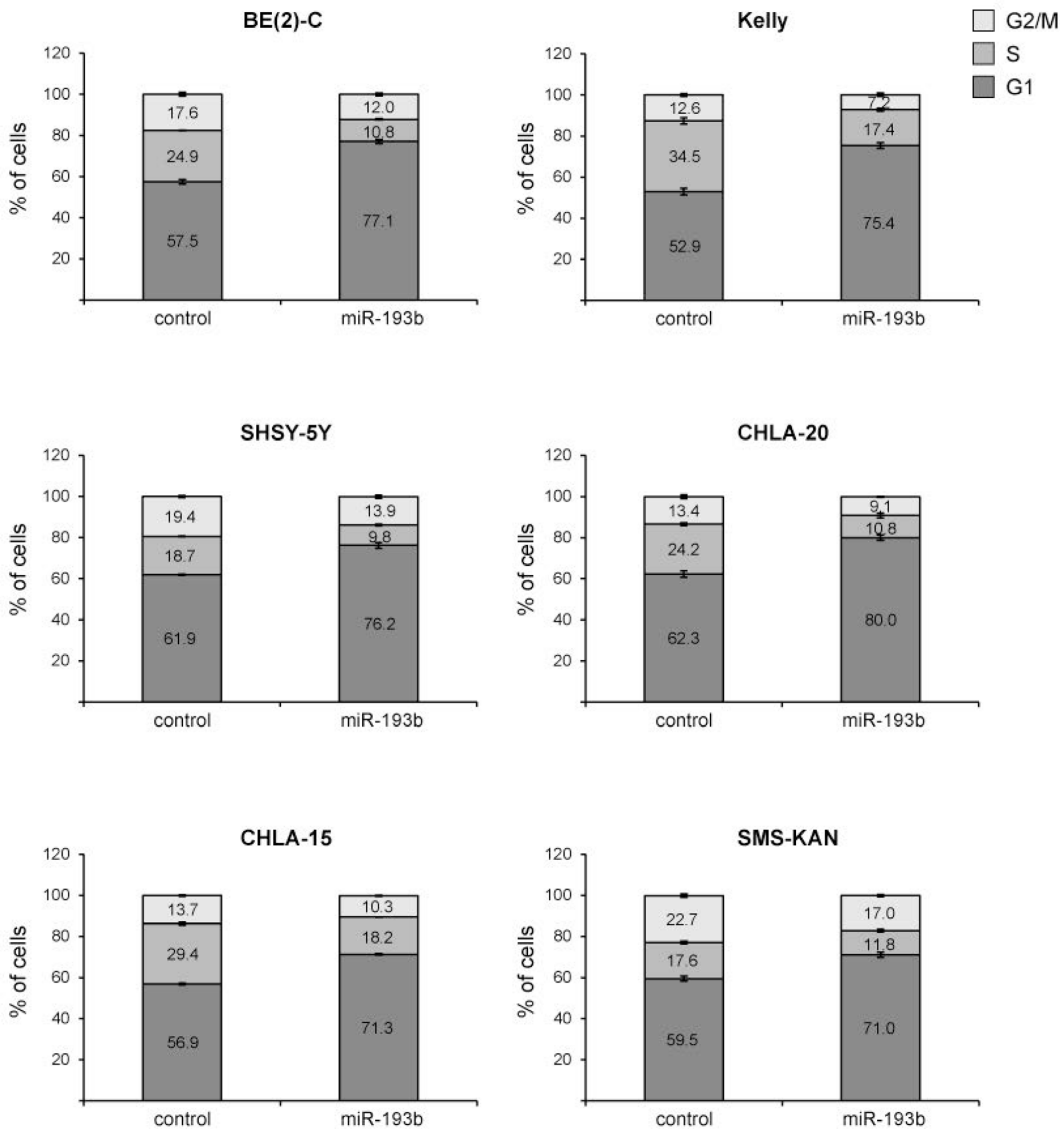
miR-193b induces a G1 cell cycle arrest in neuroblastoma Cells were transfected with control or miR-193b mimics. 24 hours after transfection, cells were fixed with ethanol and cell-cycle profiles were determined by propidium iodide incorporation and flow cytometry analysis. Results are presented as percentage of cells in a particular phase. Data are mean ± SD of one representative experiment of at least two, each performed in triplicate.

**Figure 7:**
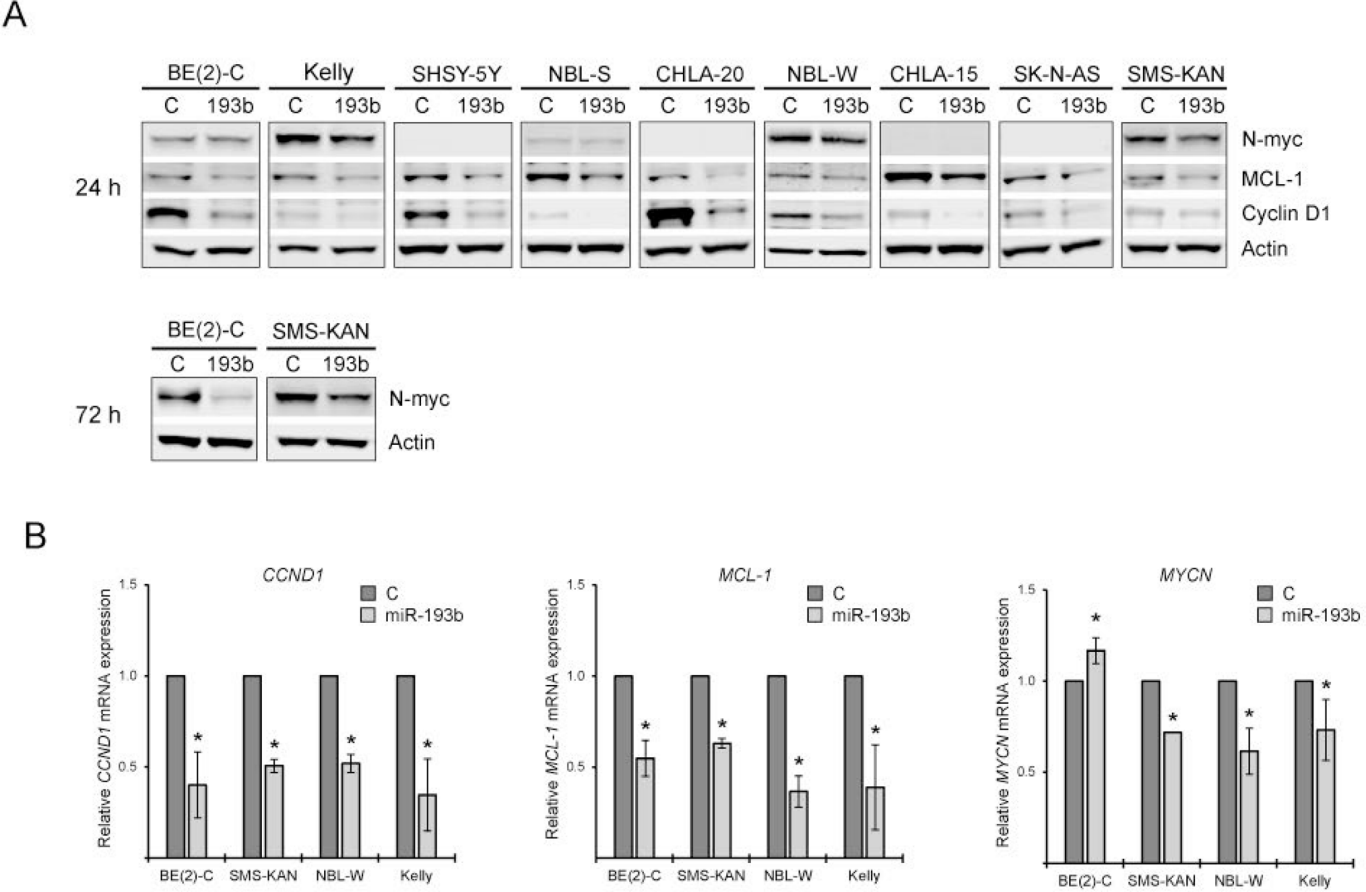
miR-193b reduces the expression of *Cyclin D1*, *MCL-1* and *MYCN* in neuroblastoma (**A**) Cells were transfected with miR-193b mimics or control mimics. 24 hours or 72 hours post-transfection, cells were harvested and protein expression was assessed by Western blot. Actin was used to demonstrate equal protein loading. The experiment was performed at least two times giving similar results, and the result from one representative experiment is shown. (**B**) Cells were transfected as in (A). 24 hours post-transfection, the total RNA was isolated, reverse transcribed into cDNA and the expression of genes were analyzed by RT-qPCR. Transcripts levels of individual genes were normalized to SDHA to allow relative quantification of gene expression relative to control-transfected cells by the DDCT method. Data are means ± SD of two independent experiments, each performed in triplicates (^*^p < 0.05 Student`s *t*-test).

### MiR-193b induces a G1 cell cycle arrest in neuroblastoma

To assess whether mir-193b-induced growth inhibition of cells is mediated via alterations in cell cycle regulation, we analyzed the cell cycle distribution profiles of mir-193b-transfected cells by flow cytometry in six of the nine cell lines. In all neuroblastoma cell lines tested, miR-193b increased the number of cells in the G1-phase of the cell cycle by 11.5–22.5% with a concomitant decrease in the number of cells in the Sphase and G2/M-phase (Figure 6). Thus, in addition to apoptosis, miR-193b decreases cell growth in neuroblastoma cell lines by inducing a G1 cell cycle arrest.

**Figure 8:**
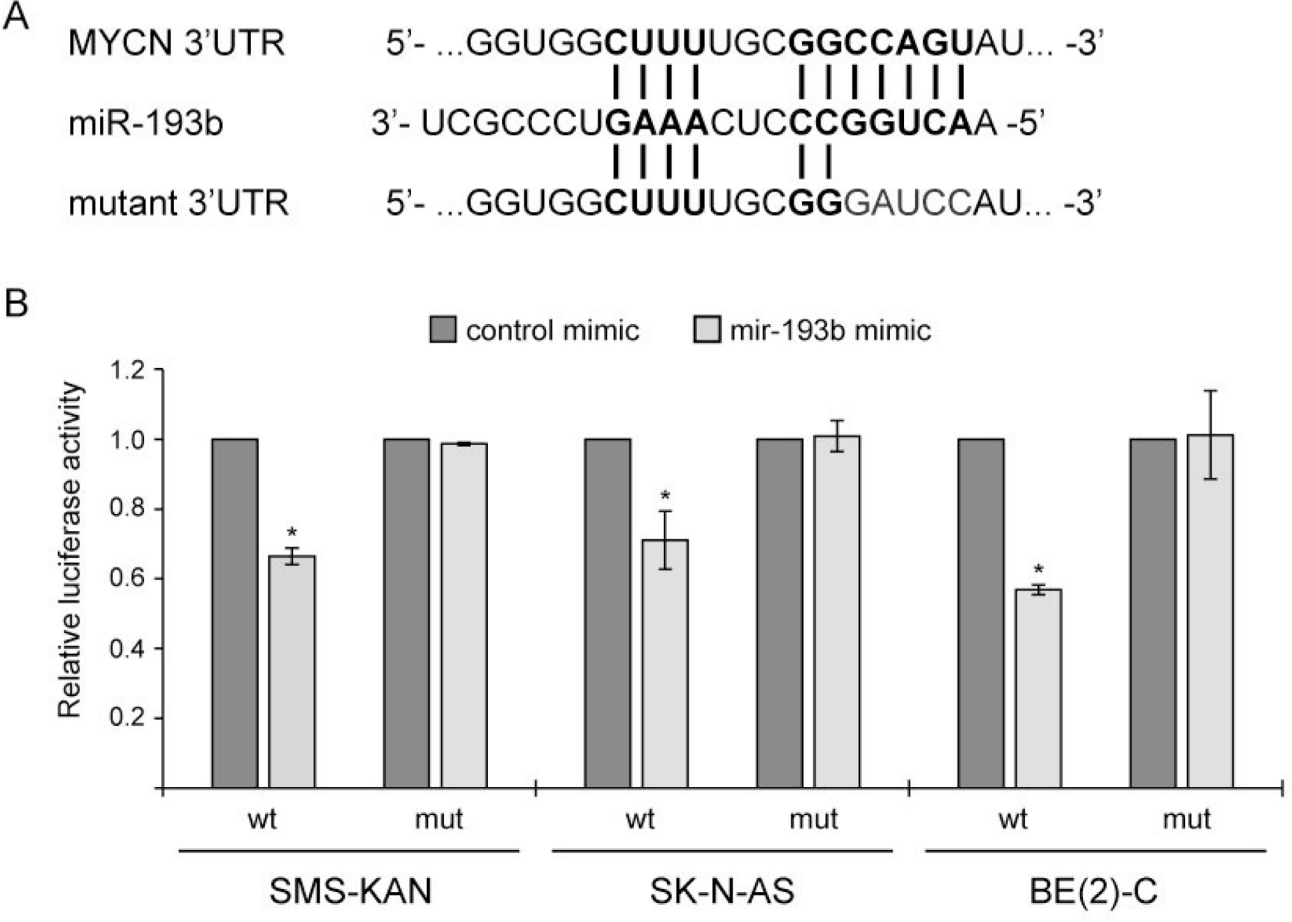
*MYCN* is a direct miR-193b target in neuroblastoma (**A**) Illustration of the putative target site of miR-193b in the 3′-UTR of *MYCN* according to TargetScan prediction and mutant *MYCN*-3`-UTR sequence. (**B**) Cells were cotransfected with miRNA mimics (either miR-193b or control mimics), a firefly luciferase report plasmids (either pMIR-Report-*MYCN*-UTR-WT or pMIR-Report-*MYCN*-UTR-MUT), and a renilla plasmids. Cells were harvested 24 hours post-transfection. Protein extracts were prepared and assayed for firefly and renilla luciferase activities. Firefly luciferase activity was normalized to renilla luciferase activity. The normalized luciferase activities of miR-193b-transfected cells were calculated relative to those of control-transfected cells (set to one). Data are means ± SD of two independent experiments, each performed in triplicate (^*^p < 0.01 Student`s *t*-test).

### MiR-193b targets *Cyclin D1*, *MCL-1* and *MYCN* in neuroblastoma

Next, we investigated the mechanism by which miR-193b may suppress cell growth in neuroblastoma cells. Both *myeloid cell leukemia 1* (*MCL-1*) and *Cyclin D1*, which are associated with apoptosis and cell cycle regulation, respectively, have been found to be direct targets of miR-193b in other cancers [34–42]. We also performed a bioinformatic analysis using TargetScan algorithm [43] to identify further potential target genes of miR-193b associated with cell cycle progression and apoptosis. The analysis revealed a highly evolutionarily conserved miR-193b binding site in the 3′-UTR of the *v-myc avian myelocytomatosis viral oncogene neuroblastoma derived homolog* (*MYCN*) oncogene (Supplementary Figure 4 ).

To test whether these genes are targeted by miR-193b in neuroblastoma, cells were transfected with either miR-193b or control mimics and the expression levels of the potential target genes were analyzed by Western blot and quantitative RT-PCR. The mRNA and protein levels of MCL-1 and Cyclin D1 were reduced upon miR-193b overexpression in all neuroblastoma cell lines tested (Figure 7A and 7B, Supplementary Figure 5). MiR-193b mimic transfection reduced *MYCN* mRNA expression in the *MYCN*-amplified cell lines SMS-KAN, NBL-W and Kelly but there was no reduction in *MYCN* mRNA expression when miR-193b mimics were introduced into BE(2)-C cells (Figure 7B). However, N-Myc protein expression was reduced in all *MYCN*-amplified cell lines tested 24 or 72 hours post-transfection (Figure 7A, Supplementary Figure 5). In BE(2)-C and SMS-KAN cells there was only a slight decrease in N-Myc protein expression 24 hours after transfection, but 72 hours post-transfection, N-Myc protein expression was lower in miR-193b transfected cells as compared to control-transfected cells (Figure 7A, Supplementary Figure 5). Thus, these results indicate that miR-193b targets *MCL-1*, *Cyclin D1* and *MYCN* in neuroblastoma cell lines.

**Figure 9:**
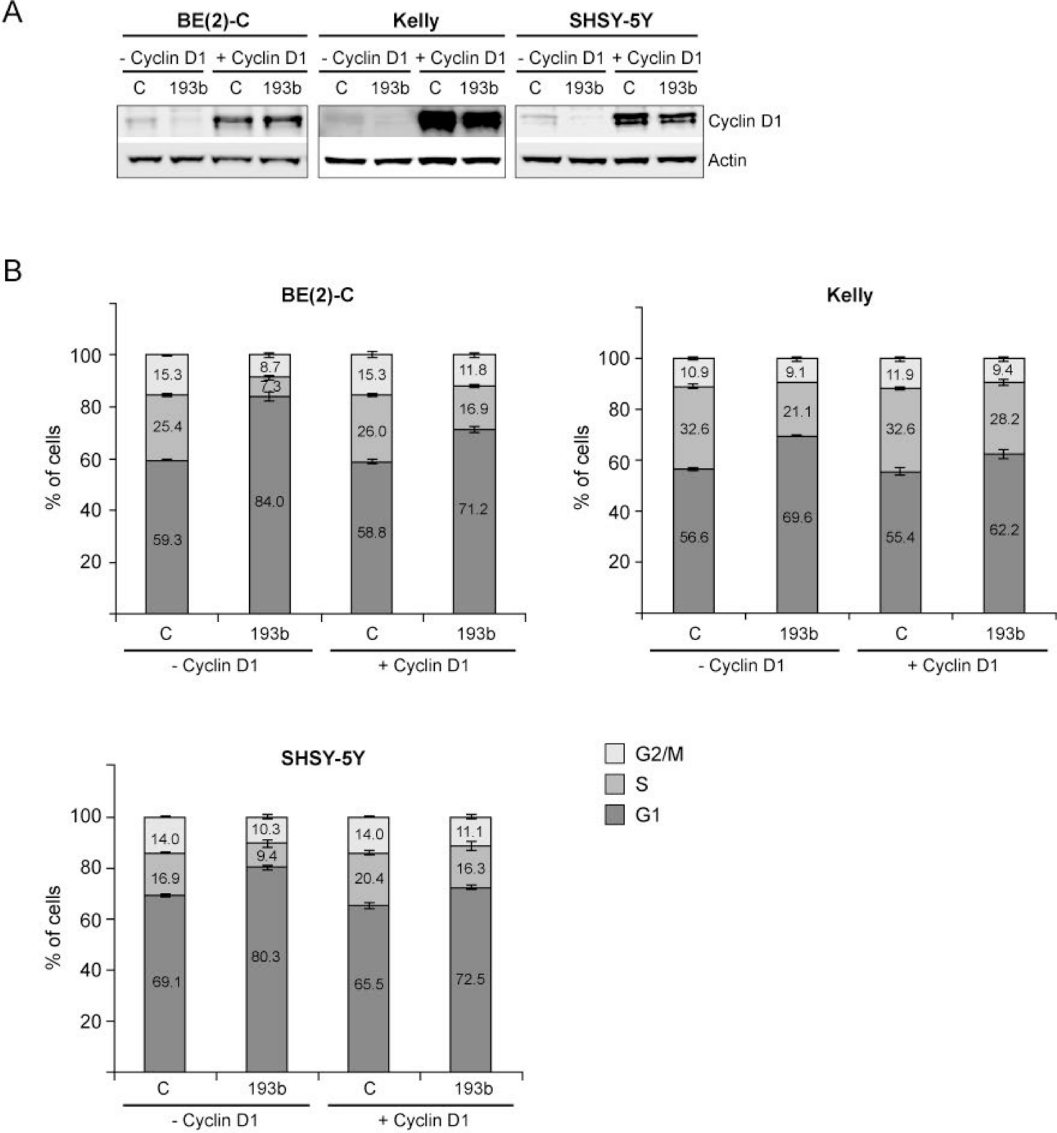
*Cyclin D1* overexpression rescues neuroblastoma cells from miR-193b-induced G1 cell cycle arrest Cells were co-transfected with control or miR-193b mimics and an empty (− Cyclin D1) or a Cyclin D1 (+ Cyclin D1) overexpression plasmid. 24 hours after transfection, cells were harvested for Western blot analysis to show Cyclin D1 overexpression (**A**) or cells were fixed with ethanol and cell-cycle profiles were determined by propidium iodide incorporation and flow cytometry analysis (**B**). Results from cell cycle analysis are presented as percentage of cells in a particular phase. Cell cycle data are mean ± SD of one representative experiment of at least two, each performed in triplicate. Western blot data show one representative experiment. Actin was used to demonstrate equal protein loading.

**Figure 10:**
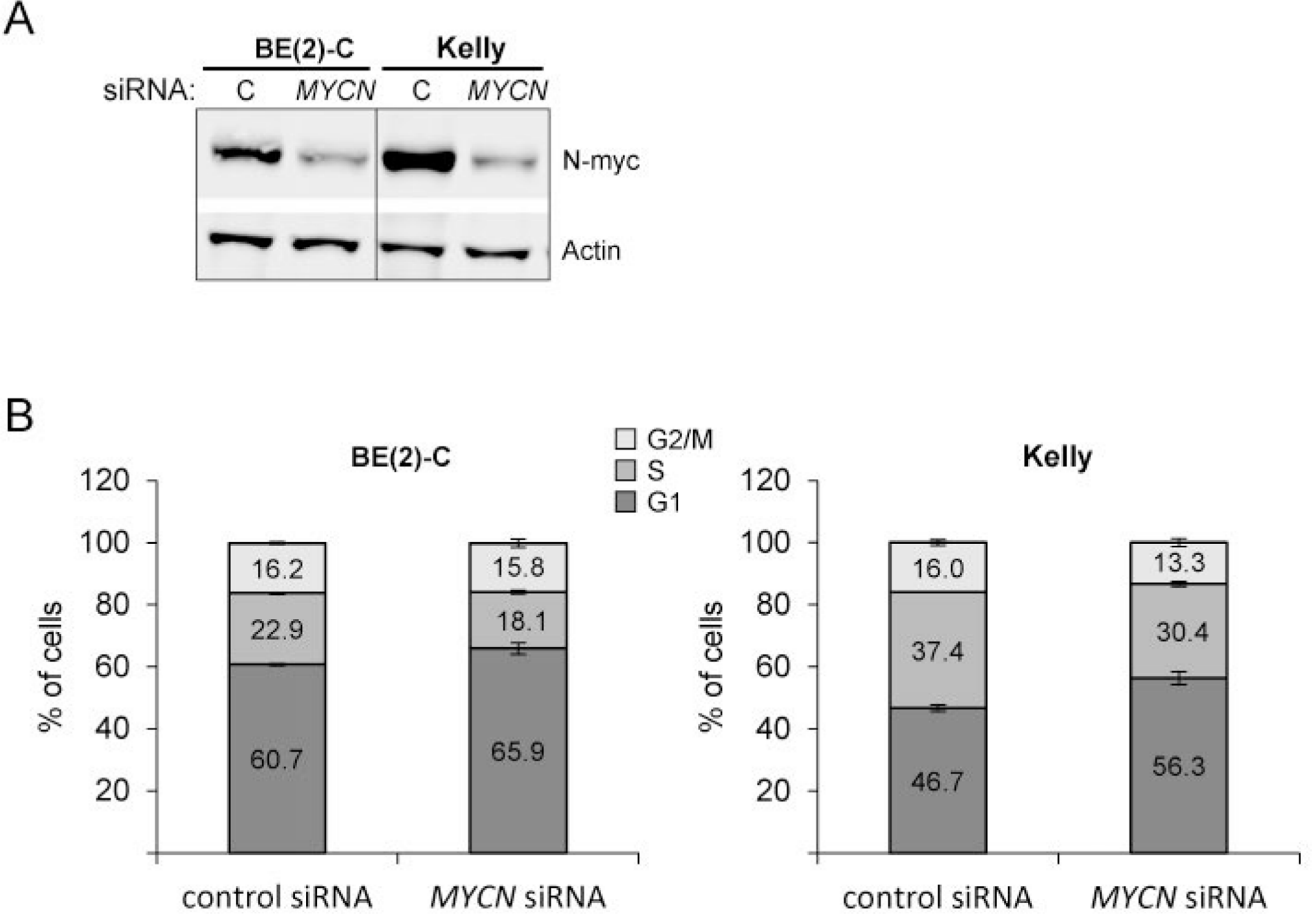
*MYCN* knockdown increases the number of cells in G1 Cells were transfected with control siRNA or *MYCN* siRNA. 24 hours after transfection, cells were harvested for Western blot analysis to show N-Myc knockdown (**A**) or cells were fixed with ethanol and cell-cycle profiles were determined by propidium iodide incorporation and flow cytometry analysis (**B**). Results from cell cycle analysis are presented as percentage of cells in a particular phase. Cell cycle data are mean ± SD of one representative experiment of at least two, each performed in triplicate. Western blot data show one representative experiment. Actin was used to demonstrate equal protein loading.

To investigate whether miR-193b directly binds to the predicted 3′UTR binding site of *MYCN*, we used a vector containing the *MYCN* 3`UTR downstream of the firefly luciferase gene (Table 1). In addition, we designed a construct containing mutations at the predicted miRNA-193b seed sequence (Figure 8A). The resulting 3′-UTR-luciferase reporter plasmids were co-transfected with control or miR-193b mimics into several neuroblastoma cell lines (*MYCN*-amplified cell lines: SMS-KAN and BE(2)-C and non-*MYCN* amplified cell line: SK-N-AS). Activity of the luciferase reporter gene carrying the wild-type *MYCN*-3-UTR was significantly lower in cells overexpressing miR-193b, while no repression of luciferase activity was observed in the reporter construct carrying the mutant *MYCN* 3`UTR (Figure 8B). These results confirm that miR-193b repress *MYCN* expression by directly binding to the 3`-UTR sequence of the *MYCN* mRNA.

**Table 1:** Plasmids used in this study

Name	Insert/property	Reference
**Expression vectors**		
pcDNA3.1-Mcl-1	MCL-1 cDNA without 3′UTR Flag-Tag (8aa) between START codon and MCL-1 cDNA	[70]
pCMV-CyclinD1	CyclinD1 cDNA without 3′UTR	[71] (Addgene # 19927)
**Luciferase reporter plasmids**		
pMIR-Report-*MYCN*-UTR-WT	*MYCN*-3′UTR reporter plasmid	[69]
pMIR-Report-*MYCN*-UTR-MUT	Mutated *MYCN*-3′UTR reporter plasmid	this study
pGL4.75[hRluc/CMV]	Renilla luciferase reporter	Promega

**Figure 11:**
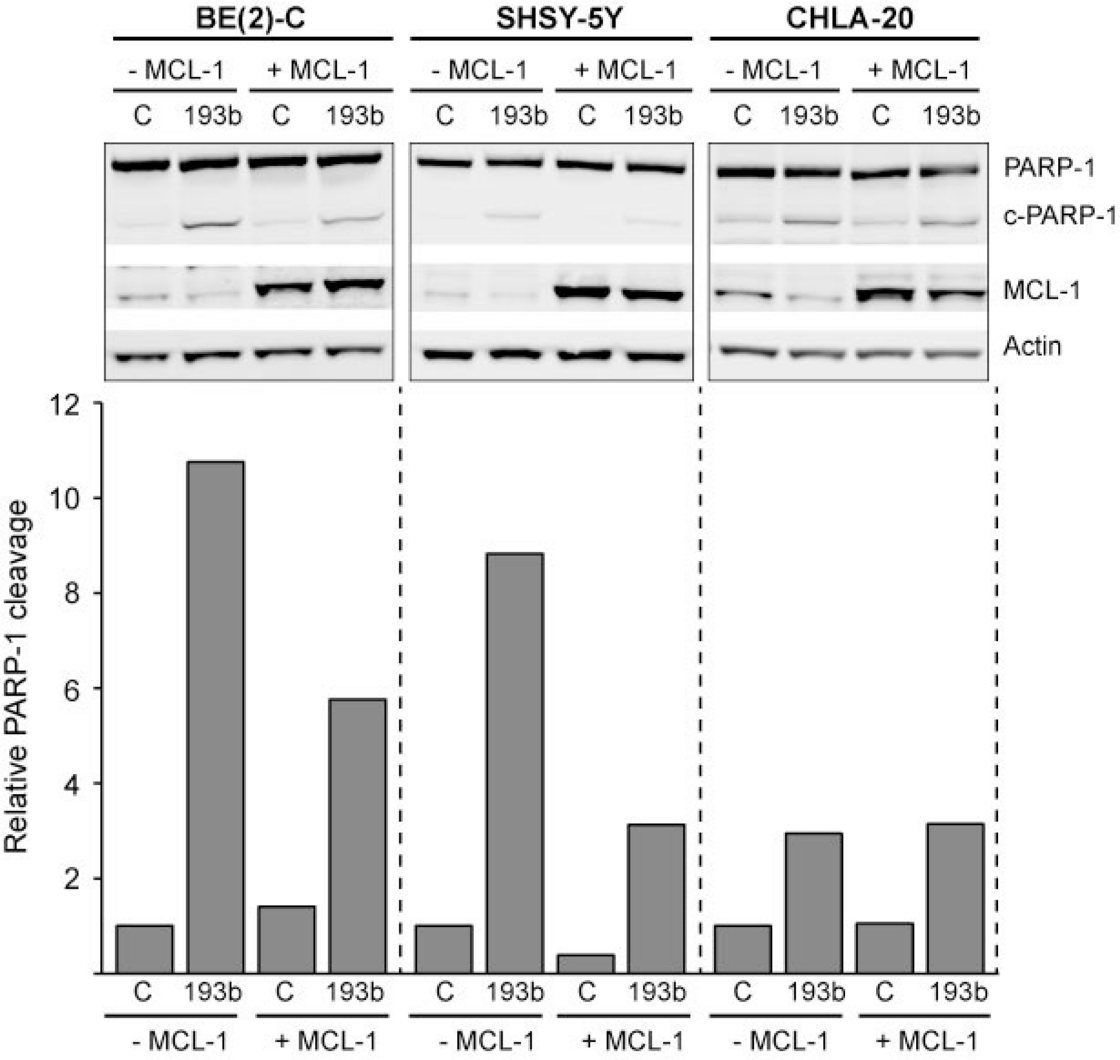
*MCL-1* overexpression partly rescues neuroblastoma cells from miR-193b-induced cell death Cells were co-transfected with control or miR-193b mimics and an empty (- Mcl-1) or an Mcl-1 (+ Mcl-1) overexpression plasmid. Cells were harvested 24 hours post-transfection. Actin was used to demonstrate equal protein loading. PARP-1 was quantified using ImageJ. Data are expressed as the ratio of cleaved PARP-1 to uncleaved PARP-1 and relative to control + empty-vector- transfected cells (set to 1). The experiment was performed at least two times giving similar results, and the result from one representative experiment is shown.

### MiR-193b induces a G1 cell cycle arrest by targeting *Cyclin D1* and *MYCN*

To address whether miR-193b induces a G1 cell cycle arrest by targeting *Cyclin D1*, cells were co-transfected with miRNA mimics and a *Cyclin D1* expression plasmid lacking the 3′UTR.

Overexpression of Cyclin D1 in Kelly, SHSY-5Y and BE(2)-C was confirmed by Western blot analysis (Figure 9A). Unfortunately, we were not able to overexpress Cyclin D1 in SMS-KAN, CHLA-15 and CHLA-20 cells (data not shown). Co-transfection of control mimics and the *Cyclin D1* overexpression plasmid had no (BE(2)-C and Kelly) or only a minor (SHSY-5Y; less than 5%) effect on cell cycle profiles (Figure 9B). *Cyclin D1* overexpression partly rescued cells from miR-193b-mediated G1 cell cycle arrest, confirming an important role of miR-193b in regulating cell cycle progression by targeting *Cyclin D1* (Figure 9B). However, the incomplete rescue by *Cyclin D1* implies that miR-193b may also modulate cell cycle progression by other mechanisms.

**Figure 12:**
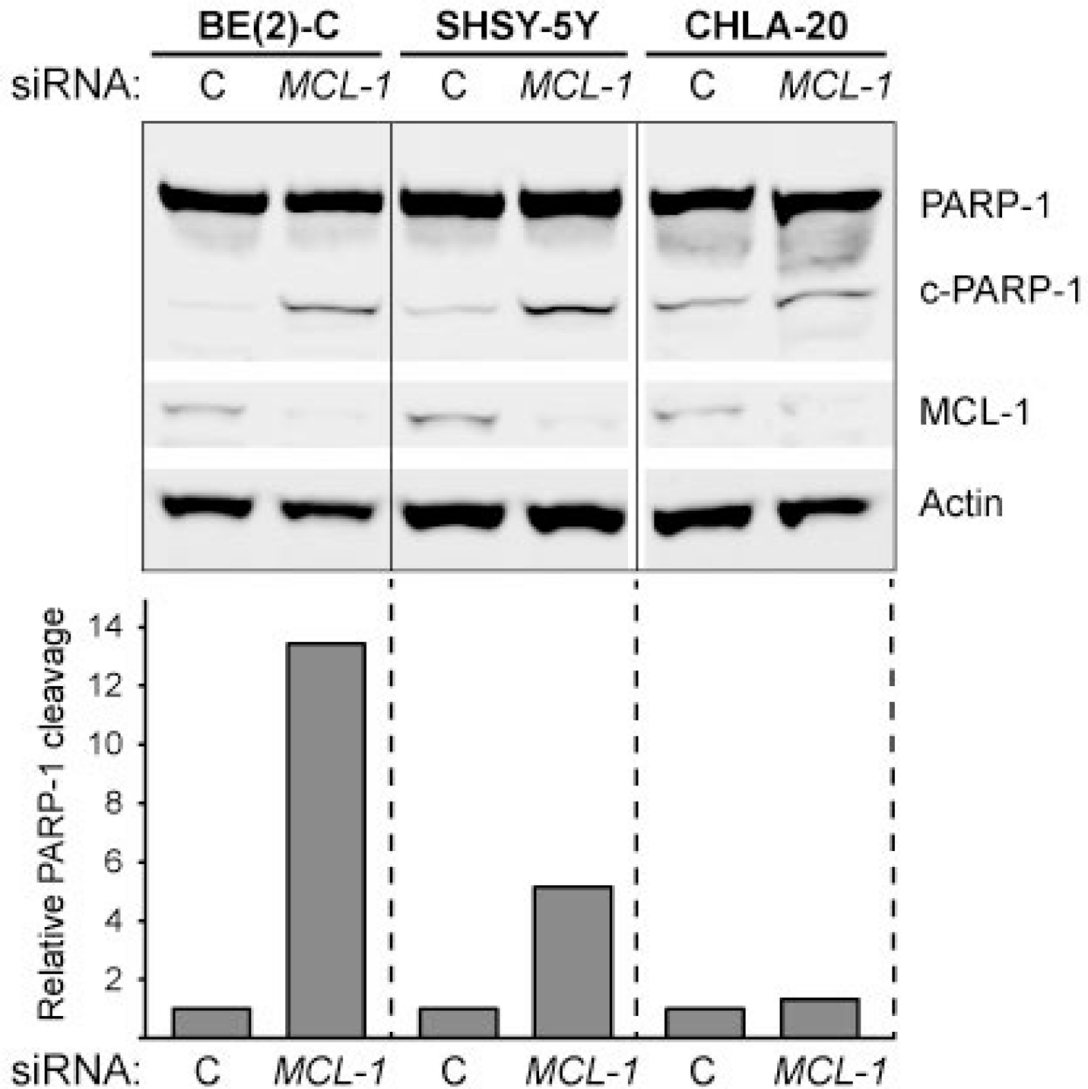
PARP-1 cleavage in response to *MCL-1* knockdown in neuroblastoma Cells were transfected with control siRNA or *MCL-1* siRNA for 24 hours. Actin was used to demonstrate equal protein loading. PARP-1 was quantified using ImageJ; data are expressed as the ratio of cleaved PARP-1 to uncleaved PARP-1 and relative to control-transfected cells (set to 1). The experiment was performed at least two times giving similar results, and the result from one representative experiment is shown.

We and others have previously shown that downregulation of *MYCN* expression induces a G1 cell cycle arrest in *MYCN*-amplified neuroblastoma cell lines including Kelly and BE(2)-C cell lines [44, 45]. To investigate whether miR-193b-mediated downregulation of *MYCN* may explain the incomplete rescue by *Cyclin D1*, BE(2)-C and Kelly cells were transfected with siRNA against *MYCN* and cell cycle profiles were analyzed. Knockdown of N-Myc was verified by Western blot analysis (Figure 10A). The results confirmed that *MYCN* knockdown causes a G1 phase arrest in Kelly and BE(2)-C cells, although the effect is considerably smaller as compared to that of miR-193b overexpression (Figure 10B). The fraction of cells in the G1 phase of the cell cycle increased from 60.7% (control siRNA) to 65.9% (*MYCN* siRNA) for BE(2)-C cells and from 46.7% (control siRNA) to 56.3% (*MYCN* siRNA) in Kelly cells transfected with 2 nM *MYCN* siRNA.

These results suggest that both *MYCN* and *Cyclin D1* may be important miR-193b targets genes mediating, at least in part, the effects of miR-193b overexpression on proliferation of neuroblastoma cells lines by inducing a G1 cell cycle arrest.

### MiR-193b induces cell death by targeting *MCL-1*

Furthermore, we analyzed whether miR-193b induces cell death by targeting *MCL-1*. For this purpose, cells were co-transfected with miRNA mimics and a *MCL-1* expression plasmid lacking the 3′UTR (Table 1). The restoration of MCL-1 expression was confirmed by Western blot analysis (Figure 11). In all cell lines tested, co-transfection of miR-193b and an empty vector resulted in increased PARP-1 cleavage, indicating cell death. Ectopic expression of *MCL-1* partly rescued SHSY-5Y and BE(2)-C cells from miR-193b-induced cell death: When transfected with both miR-193b and a vector overexpressing *MCL-1*, PARP-1- cleavage was reduced as compared to cells co-transfected with miR-193b and an empty vector (Figure 11). These results confirm that miR-193b induces cell death in SHSY-5Y and BE(2)-C cells by targeting *MCL-1*.

However, there was no rescue in CHLA-20 cells when co-transfected with the *MCL-1* expression plasmid (Figure 11). These results indicate that in addition to *MCL-1*, other miR-193b target genes are involved in mir-193b-induced cell death in neuroblastoma cells.

To verify that *MCL-1* downregulation can trigger cell death in neuroblastoma, siRNA against *MCL-1* was transfected into neuroblastoma cell lines. In concordance with the rescue experiments, *MCL-1* downregulation increased PARP-1 cleavage in BE(2)-C and SHSY-5Y whereas there was only a very modest increase in PARP-1 cleavage in CHLA-20 in response to *MCL-1* knockdown (Figure 12).

To summarize, these results indicate that the targeting of *MCL-1* by miR-193b contributes to miR-193b-induced cell death in neuroblastoma cells.

## DISCUSSION

Targeted therapy intercepting with a single oncogene is often insufficient due to preexisting or acquired resistance [46, 47]. Therefore, therapeutic approaches should simultaneously affect several targets, e.g. by using miRNAs repressing the expression of multiple targets. MicroRNAs are involved in virtually all cellular processes and exert essential roles in tumorigenesis through acting as oncogenes or tumor suppressors [6, 48]. During the last decade, development of miRNA-based anticancer therapies has received growing attention. However, picking the right miRNA exhibiting a robust phenotype and being potent and safe enough to be used as therapeutics represents a significant challenge [46].

Recent evidence suggests that miR-193b has both oncogenic and tumor suppressor functions in human cancer. MiRNA profiling studies have shown that the expression level of miR-193b is frequently downregulated in human cancer tissues as compared to non-cancerous adjacent tissues. Reduced expression levels of miR-193b as compared to (adjacent) non-cancerous cells were found in ovarian [49], hepatocellular carcinoma [34, 42], non-small cell lung cancer (NSCLC) cells [37], meningioma [50] and endometrioid adenocarcinoma [51], and also in pancreatic [52] and gastric [53, 54] cancer cells. In other cancer types, including glioma [55], multiple myeloma [56] and head and neck squamous cell carcinoma (HNSCC) [57], the expression of miR-193b has been reported to be upregulated compared to non-cancerous cells, and increased miR-193b expression levels were found in relapsed relative to non-relapsed primary HNSCC tissues [57]. Functional studies have identified both tumor suppressive and oncogenic targets of miR-193b regulating proliferation, apoptosis, differentiation, migration and invasion of various cancer cells. Thus, miR-193b regulates multiple hallmarks of cancer by targeting both oncogenes and tumor suppressors (Supplementary Figure 6). It is therefore likely that the miR-193b functions depend on multiple target genes, and both intrinsic and extrinsic factors determine whether this miRNA dominantly exerts tumor suppressive or oncogenic functions in a specific cell type.

The results of this study indicate that miR-193b exhibits tumor suppressive functions in neuroblastoma. We here demonstrated that miR-193b is generally low expressed in primary neuroblastoma samples and cell lines. A previous study revealed higher expression of miR-193b in unfavorable versus favorable neuroblastoma [18], and we recently reported increased expression of miR-193b in post-therapy neuroblastoma cell lines as compared to the matched cell lines isolated at diagnosis before treatment [21]. However, additional analyses (Figure 1B) demonstrated that miR-193b is expressed at low levels in both unfavorable neuroblastoma and in post-therapy neuroblastoma cells (Supplementary Table 1) when compared to the well-defined oncogenic miRNAs miR-92a-3p and miR-17-5p. Analysis of primary neuroblastoma samples by RT-qPCR validated low expression of miR-193b in neuroblastoma. In concordance to these findings, a previous study by Beckers *et al.* indicated that endogenous miR-193b levels do not reach the threshold to exert its tumor suppressive functions in neuroblastoma [58]. Although we and Beckers *et al.* demonstrated that MYCN is directly targeted by miR-193b, Beckers *et al.* found neither a negative correlation between miR-193b and *MYCN* expression nor miR-193b expression and MYCN activity [58]. Thus, the direct miR-193b target *MYCN* is insufficiently counteracted by low endogenous miR-193b expression levels in neuroblastoma. Our *in vitro* data indicate that correcting these deficiencies by miR-193b replacement therapy, effectively represses *MYCN* and two further important oncogenic miR-193b targets, namely *MCL-1* and *cyclin D1* resulting in the activation of anti-proliferative and pro-apoptotic pathways. We thereby showed that the increase in miR-193b levels upon mimic transfection significantly augments the antagonizing functions of miR-193b beyond the threshold at which this miRNA becomes dominantly tumor suppressive. Thus, oncogenic pathways, which are insufficiently counteracted by endogenous miR-193b levels, are effectively repressed upon miR-193b mimic transfection. Further studies are required to determine the extent to which *cyclin D1*, *MYCN* and *MCL-1* are actually regulated by endogenous levels of miR-193b.

### MicroRNA-193b Induces a G1 Cell Cycle Arrest by Targeting Cyclin D1 and MYCN

Previous studies have shown that deregulation of the cell cycle, in particular G1 entry checkpoint dysregulation, appears to be an important factor in driving neuroblastoma tumorigenesis [59–61]. Several cell cycle regulators, especially those within the cyclin D1/CDK4/CDK6/RB pathway, are hyperactive in neuroblastoma [61]. Recent *in vitro* and *in vivo* studies on the therapeutic utility of inhibitors targeting the cyclin D1-associated kinases CDK4/CDK6 revealed promising results in various cancer types including neuroblastoma [59, 61].

The present study demonstrates that introduction of miR-193b into neuroblastoma cell lines results in a G1 cell cycle arrest via downregulation of *cyclin D1*. However, overexpression of *cyclin D1* only partly rescue miR-193b-mediated G1 cell cycle arrest pointing to additional miR-193b target genes whose inhibition directly or indirectly induces a G1 cell cycle arrest.

Our data indicate that the oncogene *MYCN* is one such target regulated by mir-193b. We have previously shown that downregulation of *MYCN* induces a G1 cell cycle arrest in *MYCN*-amplified neuroblastoma cell lines [44]. In concordance with this, we demonstrated that siRNA-mediated knockdown of *MYCN* increases the proportion of *MYCN*-amplified neuroblastoma cells in the G1 phase of the cell cycle.

Based on these results, we conclude that the observed anti-proliferative effect of miR-193b in neuroblastoma is mediated, at least in part, through targeting both *cyclin D1* and *MYCN*, two oncogenes with essential roles in neuroblastoma tumorigenesis and thereby attractive therapeutic targets.

### MicroRNA-193b Induces cell death in neuroblastoma

The functions of miR-193b in the control of the cell cycle are complemented by functions regulating cell death. Apoptosis is the best-characterized mechanism of programmed cell death in which activation of the executioner caspase cascade promotes cell shrinkage, membrane blebbing, chromatin condensation, DNA fragmentation, and finally the formation of apoptotic bodies and cell death [62]. In recent years, a number of non-canonical cell death mechanisms, which are often caspase-independent, have been described. These include caspase-independent apoptosis (CIA), necrosis, mitotic catastrophe, ferroptosis, paraptosis, and pyroptosis [62].

To investigate whether the anti-proliferative effect of miR-193b is also mediated through the induction of apoptosis, we analyzed caspase-3/7 activity and poly(ADP-ribose)-polymerase (PARP)-1 cleavage, which are hallmarks of apoptosis. We found that miR-193b significantly increases both caspase-3/7 activity and PARP-1 cleavage in six of nine investigated neuroblastoma cell lines within 24 hours. Interestingly, while miR-193b only slightly increased caspase-3/7 activity in CHLA-15 cells (1.26 fold), it significantly increased PARP-1 cleavage in this cell line within 24 hours post-transfection, indicating that miR-193b may also induce caspase-independent cell death in neuroblastoma. In SK-N-AS cells, miR-193b-mediated PARP-1 cleavage was not observed before 72 hours post-transfection, suggesting that miR-193b does not directly trigger cell death in this cell line, but rather induces cell death indirectly by modulating signaling cascades, which leads to secondary (delayed) cell death. Thus, these findings suggest that miR-193b may induce directly or indirectly (secondary) cell death in neuroblastoma by several distinct mechanisms.

A number of studies have revealed that miR-193b triggers apoptosis in various cancer cells via downregulation of *MCL-1* [35, 39, 41, 42]. We demonstrated that miR-193b overexpression reduces *MCL-1* mRNA and protein expression in neuroblastoma cells. However, while *MCL-1* overexpression could partly rescue SK-N-BE(2) and SHSY-5Y cells from miR-193b-mediated apoptosis, it could not rescue CHLA-20 cells.

Previous studies established that neuroblastoma cells are primed to death through sequestration of BIM by either MCL-1 or BCL-2 [63, 64]. CHLA-20 is a well-defined BCL-2-primed cell line, whereas SK-N-SH (SHSY-5Y is a subclone of this cell line [65]) and SK-N-BE(2) cells are MCL-1-primed [63]. Goldsmith *et al.* demonstrated that BCL-2-primed cell lines are significantly less sensitive to MCL-1 inhibition by AT-101 (a BH3 mimetic that also binds to BCL-2 but less potently than to MCL-1) as compared to MCL-1-primed cell lines [64]. Concordantly we demonstrated that, while *MCL-1* knockdown by siRNA effectively induces PARP-1 cleavage in MCL-1-primed SHSY-5Y and SK-N-BE(2) cells, it does not trigger PARP-1 cleavage in the BCL-2-primed cell line CHLA-20. These findings support a selective dependence of neuroblastoma cells on either BCL-2 or MCL-1, and explain why *MCL-1* overexpression could rescue BE(2)-C and SHSY-5Y cells, but not rescue the BCL-2-primed neuroblastoma cell line CHLA-20 from miR-193b-induced apoptosis. The mechanisms through which miR-193b may induce apoptosis in BCL-2-primed cell lines remain to be determined.

To summarize, our date provide evidence that miR-193b is capable of inducing cell death by several, distinct mechanisms. Simultaneously targeting several signaling pathways activating cell death may increase the antitumor efficiency of miR-193b-based anticancer therapy and reduce acquired resistance to miR-193b-mediated cell death. Further studies are needed to fully understand the mechanisms by which miR-193b exerts its tumor suppressive function in neuroblastoma.

In summary, miR-193b overexpression induces a cell cycle arrest and apoptosis in neuroblastoma by reducing the expression of *MYCN*, *cyclin D1* and *MCL-1*, three important oncogenes in neuroblastoma whose inhibition by inhibitors have shown promising results in preclinical testing [59, 61, 66, 67]. Our data suggest that miR-193b may be a promising strategy to treat high-risk neuroblastoma resistant to conventional anticancer agents. This is especially supported by the finding that this miRNA has anti-tumor properties in all neuroblastoma cell lines tested. MiR-193b induced a cell cycle arrest and cell death independent on risk factors such as amplification of the *MYCN* oncogene or p53 functionality (Supplementary Table 2). Even cell lines from high-risk neuroblastoma such as BE(2)-C and SK-N-AS, which are highly resistant to most of the currently used neuroblastoma treatment strategies, are sensitive to the treatment with miR-193b. There are clearly certain limitations to our study. First of all, the analyses of mir-193b expression in neuroblastoma tumors were performed in a limited number of tumors. A larger cohort of tumors should be used to validate our finding. And secondly, all the functional analyses were performed in neuroblastoma cell line models. Although cell lines are useful and easily available for *in vitro* analyses, animal models are needed to explore the effect of miR-193b on neuroblastoma tumors *in vivo*.

## MATERIALS AND METHODS

### Tumor specimens

Previously published expression data by us from primary neuroblastoma tumor specimens representing the whole disease spectrum was reanalyzed for this study [18, 33]. In short, a panel of 69 tumor samples was analyzed by RT-qPCR, and 10 different tumor samples were analyzed by RNA-Seq. The expression of miR-193b in comparison to tumor suppressor, or oncomiRs was analyzed and statistically evaluated. For technical details please refer to our previous publications.

### Cell lines

CHLA-20 and CHLA-15 cells were grown in Iscove's modified Dulbecco's medium supplemented with 20% fetal bovine serum, 4 mM L-Glutamine and 1× ITS (5 μg/ml insulin, 5 μg/ml transferrin, 5 ng/ml selenous acid). The remaining cell lines were grown in RPMI-1640 supplemented with 10% fetal bovine serum and 2 mM L-Glutamine (final concentrations). An overview of the genetic abnormalities for cell lines used in this study is shown in Supplementary Table 2.

All cell lines were cultured at 37° C in a humidified incubator containing a 5% CO_2_ atmosphere. The identity of all cell lines was authenticated by short tandem repeat analysis at the Center of Forensic Genetics, The Arctic University of Norway – UiT, Norway. Cells were tested and confirmed negative for mycoplasma contamination.

### Transient transfections of miRNAs, plasmids and siRNA

All cell lines were transiently transfected immediately after seeding using Lipofectamine2000 (ThermoFisher Scientific) according to manufacturer's instructions.

To analyze the effect of miR-193b mimics on neuroblastoma cell lines, 25 nM of control mimics or miR-193b-3p mimics (GenePharma) were transfected into neuroblastoma cell lines.

For rescue experiments, cells were co-transfected with 500 or 1 μg of an overexpression plasmid (Table 1) and 40 nM control or miR-193b mimics.

Small inhibitory RNAs (siRNA; OnTargetPLUS SmartPool) directed against *MCL-1* (siMcl-1) and control siRNA were purchased from Dharmacon. SiRNA directed against *MYCN* (si*MYCN*, Hs_*MYCN*_6 FlexiTube siRNA) and negative control siRNA (AllStars Negative Control siRNA) were purchased from Qiagen. Cells were transfected with 20 nM (siMcl-1) or 2 nM (si*MYCN*) siRNA.

### Cell viability assay

Cell viability was evaluated using alamarBlue (ThermoFisher Scientific) according to manufacturer's recommendation. Briefly, 1/10th volume of alamarBlue^®^ reagent was directly added to cells in culture medium and cells were incubated at 37° C for three hours. One hundred microliter of supernatant were then collected from each well, transferred to a black-walled 96-well plate and fluorescence was monitored at 540 nm excitation wavelength and 590 nm emission wavelength in a microplate reader (CLARIOstar, BMG LABTECH). Cell viability was calculated as a percentage of control-transfected cells.

### xCELLigence

Cells were seeded into a 16-well E-Plate (harboring a high-density gold electrode array to measure electrical impedance) and transfected with control or miR-193b mimics immediately after seeding as described previously. The culture medium was changed 72–96 h post-transfection. Cell proliferation was monitored continuously and recorded as a delta cell index (DCI) every 30 min for 168 hours by the xCELLigence Real-Time Cell Analyzer (RTCA)-DP system (ACEA Biosciences). The DCI was defined as the cell index (CI) at a given time point plus a Delta value. The Delta value is the difference between a reference value (=1) and the cell index at the Delta time point (CI_1_ = CI one hour post-transfection): DCI = CI + (1−CI_1_).

### Cytotoxicity assay

Cytotoxicity was analyzed using the MultiTox-Fluor Multiplex Cytotoxicity Assay (Promega) according to manufacturer`s recommendation. Briefly, one volume of the MultiTox-Fluor reagent was directly added to cells in culture and incubated at 37° C for 30 min. One hundred microliter of supernatant was then collected from each well, transferred to a white-walled 96-well plate and fluorescence was measured in a microplate reader (CLARIOstar, BMG LABTECH). Amounts of cells with intact (living) and disrupted cell membranes (dead) were measured at 400 nm and 485 nm excitation wavelength or 505 and 520 nm emission wavelength, respectively. Normalization involved two steps. First, the number of viable cells was normalized to the number of dead cells, and then the ratio of viable to dead cells of miR-193b-transfected cells was calculated relative to that of control-transfected cells for which the ratio was set to 100%. A decrease in the ratio of viable to dead cells indicates cytotoxicity. Apoptosis assay

Caspase-3/7 activity as an indicator of apoptosis was determined using the Caspase-Glo 3/7 assay (Promega) according to manufacturer`s recommendation. Briefly, one volume of the Caspase-Glo 3/7 reagent was directly added to cells in culture and incubated at room temperature for 90 minutes. One hundred microliter of supernatant were then collected from each well, transferred to a white-walled 96-well plate and luminescence was measured in a microplate reader (CLARIOstar, BMG LABTECH).

### Cell cycle analysis

To monitor cell cycle profiles, cells were seeded into 25 cm^2^ culture flasks and transfected immediately after seeding as described previously. 24 hours post-transfection, the medium was collected and the cells were removed from plates using trypsin. Cells in suspension were then added to the collected medium, centrifuged at 200 g and washed twice with phosphate-buffered saline (PBS). The cells were fixed in ice-cold 70% ethanol. After incubation at 4° C overnight, the fixed cells were washed twice with PBS and resuspended in 50 μl PBS containing 100 μg/ml RNase. 200 μl PBS with 50 μg/ml propidium iodide (PI) was added to each sample, incubated in the dark at room temperature for 30 minutes and stored on ice until analyzed by flow cytometry (BD LSRFortessa™ cell analyzer, BD Bioscience). Cell-cycle data were analyzed with the FlowJo 7.6.5 software using the Watson model for cell-cycle evaluation.

### Real-time (RT)-PCR

Total RNA was isolated using TRIzol (ThermoFisher Scientific) according to manufacturer's instructions. Purity and concentration of TRIzol-isolated RNA used for reverse transcription was estimated photometrically by calculating the ratio of absorbance at 260 nm to the absorbance at 280 nm, as well as the ratio of absorbance at 260 nm to that of 230 nm using a NanoDrop™ 1000 spectrophotometer (ThermoFisher Scientific).

Complementary DNA (cDNA) from miRNAs was synthesized from isolated total RNA using the miScript II RT Kit (Qiagen). In short, 1 μg of isolated total RNA was brought to a total volume of 12 μl with deionized water. 2 μl of miScript Reverse Transcriptase Mix and 2 μl of miScript Nucleics Mix diluted in 4 μl of 5 × miScript HiSpec buffer were added to each sample. The reverse transcription was performed at 37° C for 60 minutes followed by 95° C for 5 minutes to terminate the reaction. The resulting cDNA was diluted with deionized water to achieve a concentration of 1 ng/μl and stored at −20° C until used in the real-time polymerase chain (RT-PCR) reaction. The miScript SYBR^®^ Green PCR Kit (Qiagen) was used for quantitative RT-PCR. The PCR was performed in a 10 μl reaction volume according to the following protocol: Each 10 μl reaction was composed of 1 μl of cDNA equivalent to 1 ng of cDNA, 2 μl of water, 5 μl of QuantiTect SYBR Green, 1 μl of 10 x Universal primers and 1 μl of 10 × specific miScript primers. The following miScript Primer Assays (Qiagen) were used: Hs_miR-193b_3 (MS00031549), Hs_miR-34a_1 (MS0003318), Hs_miR-92_1 (MS0006594) and Hs_RNU6-2_11 (MS00033740) that was used as an internal control for normalization.

cDNA from mRNAs was synthesized from 1 μg of total isolated RNA by Maxima Reverse Transcriptase (ThermoFisher Scientific) using oligo dT primer: 1 μg of isolated RNA and 1 μl of both oligo dT primer (20 μM) and dNTP (10 mM each) was brought to a total volume of 15.75 μl with deionized water. The mixture was incubated for 5 minutes at 65° C to break down secondary structures and subsequently incubated at 4° C until the reverse transcription mix was added. 0.25 μl Maxima Reverse Transcriptase diluted in 4 μl 5 × RT-Buffer was added to each sample. The reverse transcription was performed at 60° C for 30 minutes followed by 85° C for 5 minutes to terminate the reaction. The resulting cDNA was diluted in 80 μl deionized water to achieve a concentration of 10 ng/μl and stored at −20° C until used in the RT-PCR reaction. The RT-PCR was performed in a 10 μl reaction volume according to the following protocol: Each 10 μl reaction was composed of 1 μl cDNA equivalent to 10 ng cDNA, 3.2 μl water, 5 μl POWER SYBR (ThermoFisher Scientific) and 0.4 μl of 10 μM sense and antisense primer (Table 2).

**Table 2:** Oligonucleotides used in this study

Name	Orientation	Sequence (5′ to 3′)
**Site directed mutagenesis**		
*MYCN*-3′UTR-193b-f	forward	ATGAGAGGTGGCTTTTGCGGGATCCA TTAGACTGGAAGTTCATAC
*MYCN*-3′UTR-193b-r	reverse	GTATGAACTTCCAGTCTAATGGATC CCGCAAAAGCCACCTCTCAT
**RT-qPCR**		
*MCL-1*-f	forward	GATGCAGCTTTCTTGGTTTATGG
*MCL-1*-r	reverse	GATGCAGCTTTCTTGGTTTATGG
*CCND1*-f	forward	CCGTCCATGCGGAAGATC
*CCND1*-r	reverse	ATGGCCAGCGGGAAGAC
*MYCN*-f	forward	ACACCCTGAGCGATTCAGAT
*MYCN*-r	reverse	TTCTCCACAGTGACCACGTC
*SDHA*-f	forward	CTGATGAGACAAGATGTGGTG
*SDHA*-r	reverse	CAATCTCCCTTCAATGTACTCC

Oligonucleotides were purchased from Sigma-Aldrich or Invitrogen.

Amplifications were carried out according to manufacturer's recommendations using the LightCycler 96 SW 1.1 (Roche). To confirm amplification specificity, a melt curve was generated after the completion of the amplification reaction. Expression levels of mRNAs were evaluated using the 2^–ΔΔCT^ comparative cycle threshold method [68]. To determine differential expression of mRNAs between control and miR-193b-transfected cells, mRNA expression levels of miR-193b-transfected cells were calculated relative to mRNA expression levels of control-transfected cells whose mean mRNA expression was set to 1.

### Western blot analysis

Cells were harvested by standard procedure. Briefly, the culture medium was collected and the cells were washed with PBS. To dissociate adhesive cells from the culture plates, trypsin was added. Cells in suspension were then added to the collected culture medium and centrifuged at 200 g for 5 minutes. After an additional washing step with PBS, cells were lysed in RIPA buffer (50 mM Tris-HCl pH 8, 150 mM NaCl, 1% NP-40, 0.5% sodium deoxycholate, 0.1% SDS) supplemented with 1× Protein Inhibitor Cocktail (Roche) and 1 mM dithiothreitol (DTT). Protein concentrations were determined using the DC™ protein assay kit (Bio-Rad) according to the manufacturer's recommendation. The total protein concentration was calculated based on a bovine serum albumin (BSA) standard curve using a microplate reader (CLARIOstar, BMG LABTECH).

20–40 μg of protein in NuPAGE^®^ LDS Sample Buffer (ThermoFisher Scientific) was separated on NuPAGE^®^ Novex 4–12% Bis-Tris precast polyacrylamide gels (ThermoFisher Scientific) and subsequently electroblotted onto Immobilon-FL PVDF (Millipore). PVDF membranes were blocked in Odyssey blocking buffer (LI-COR Biosciences) for 1 hour at room temperature, and incubated with primary antibodies overnight at 4° C. The membrane was treated with secondary antibodies goat anti-rabbit IRDye800CW (1:5000) (Rockland Immunochemicals) and goat anti-mouse Alexa Fluor 680 (1:5000) (ThermoFisher Scientific) for 1 hour at room temperature. Antibody binding was detected using the Odyssey CLx infrared imaging system (Li-Cor). Primary antibody specifications are specified in Table 3.

**Table 3:** Primary antibodies used in this study

Antibody	Epitope	Origin	Dilution	Supplier
MCL-1 (S-19)	Human MCL-1	Rabbit, polyclonal	1:1000	Santa Cruz Biotechnology
Cyclin D1 (H-295)	Human Cyclin D1	Rabbit, polyclonal	1:200	Santa Cruz Biotechnology
PARP-1 (9542)	Human full length and cleaved PARP-1	Rabbit, polyclonal	1:1000	Cell Signalling Technology
N-Myc (B8.4.B)	Human N-Myc	Mouse, monoclonal	1:400	Santa Cruz Biotechnology
Actin (ab3280)	Human actin	Mouse, monoclonal	1:1000	Abcam

### Dual-luciferase reporter assay

The pMIR-Report *MYCN*-UTR vector containing the wildtype putative target site of *MYCN* 3′UTR has been previously generated by our group [69]. QuikChange Multi Site-directed Mutagenesis Kit (Agilent Technologies) was used to mutate the putative miR-193b seed sequence. The sequences of all constructs were confirmed by bidirectional sequencing. Primers used for cloning and site-directed mutagenesis are listed in Table 2.

Cells were co-transfected with 20 ng pGL4.75 [hRluc/CMV] (Promega), 40 nM of either control mimics or miR-193b mimics, and 100 ng of either wild-type *MYCN*-3-UTR construct (pMIR-Report-*MYCN*-UTR-WT) or the mutant variant (pMIR-Report-*MYCN*-UTR-MUT) using Lipofectamine2000. At 24 hours post-transfection, renilla and firefly luciferase activities were analyzed using the Dual-Luciferase Reporter Assay (Promega) according to the manufacturer's instructions. Normalization included two steps: first, the firefly luciferase activity was normalized to the renilla luciferase activity, and second, the luciferase activities of miR-193b-transfected cells were calculated relative to control-transfected cells that were set to one.

### Statistical analysis

Statistical analyses were carried out using R (http://www.r-project.org). Statistical differences between means were determined using the parametrical Student`s *t*-test, or where not-applicable, the non-parametric Man-Whitney-*U* test.

Steffen Fuchs is participant in the BIH Charité Clinician Scientist Program funded by the Charité – Universitätsmedizin Berlin and the Berlin Institute of Health.

## SUPPLEMENTARY MATERIALS FIGURES AND TABLES


