# Supplementary material for: MicroRNA-193b-3p represses neuroblastoma cell growth via downregulation of Cyclin D1, MCL-1 and MYCN
Source: Oncotarget. 2018 Apr 6;9(26):18160–79. doi: 10.18632/oncotarget.24793 (PMC5915064; doi:10.18632/oncotarget.24793)
Supplement: Supplementary file 1 [file oncotarget-09-18160-s001.pdf]

MicroRNA-193b-3p represses neuroblastoma cell growth via downregulation of *Cyclin D1*, *MCL-1* and *MYCN*

SUPPLEMENTARY MATERIALS

Supplementary Table 1: Low expression of miR-193b in neuroblastoma cell lines. Total RNA was isolated from cell lines, enriched for small RNAs and small RNA sequencing was performed according to Roth *et al.* 2016. See Supplementary_Table_1

Supplementary Table 2: Genetic characteristics of neuroblastoma cell lines used in this study

Cell line	Stage	Origin	Treatment	MYCN-amplified	p53	1p del	11q del	17q gain	References
BE(2)-C	4	Bone marrow (primary: unknown)	YES	YES	MUT	YES	NO	NO	[1, 2]
Kelly	4	Bone marrow	YES	YES	MUT	YES	YES	YES	[3, 4]
SHSY-5Y*	?	Bone marrow (primary: thorax)	YES	NO	WT	NO	NO	YES	[1, 5, 6]
CHLA-20	4	Primary, progressive	YES	NO	WT	?	?	?	[7, 8]
NBL-W	4S	Adrenal (primary)	NO	YES	WT	YES	?	NO	[1, 9]
CHLA-15	4	Primary	NO	NO	WT	?	?	?	[7, 10]
NBL-S	3	Adrenal (primary)	NO	NO	WT	NO	?	NO	[1, 11]
SK-N-AS	4	Bone marrow (primary: adrenal)	YES	NO	MUT	YES	YES	NO	[1, 12, 13]
SMS-KAN	4	Pelvis (primary)	NO	YES	WT	YES	YES	YES?	[1, 14, 15]

*parental cell line: SK-N-SH

1. Thiele CJ. Neuroblastoma Cell Lines, in Neuroblastoma, J.H.C. Culture. 1998: Lancaster. UK. 21–53.
2. Tweddle DA, Malcolm AJ, Bown N, Pearson AD, Lunec J. Evidence for the development of p53 mutations after cytotoxic therapy in a neuroblastoma cell line. Cancer Res. 2001; 61:8–13.
3. Gogolin S, Ehemann V, Becker G, Brueckner LM, Dreidax D, Bannert S, Nolte I, Savelyeva L, Bell E, Westermann F. CDK4 inhibition restores G(1)-S arrest in MYCN-amplified neuroblastoma cells in the context of doxorubicin-induced DNA damage. Cell Cycle. 2013; 12:1091–104.
4. Piskareva O, Harvey H, Nolan J, Conlon R, Alcock L, Buckley P, Dowling P, Henry M, O'Sullivan F, Bray I, Stallings RL. The development of cisplatin resistance in neuroblastoma is accompanied by epithelial to mesenchymal transition *in vitro*. Cancer Lett. 2015; 364:142–55.
5. Djos A, Fransson S, Kogner P, Martinsson T. Aneuploidy in neuroblastoma tumors is not associated with inactivating point mutations in the STAG2 gene. BMC Med Genet. 2013; 14:102.
6. Tweddle DA, Malcolm AJ, Cole M, Pearson AD, Lunec J. p53 cellular localization and function in neuroblastoma: evidence for defective G(1) arrest despite WAF1 induction in MYCN-amplified cells. Am J Pathol. 2001; 158:2067–77.
7. Keshelava N, Seeger RC, Groshen S, Reynolds CP, Drug resistance patterns of human neuroblastoma cell lines derived from patients at different phases of therapy. Cancer Res. 1998; 58:5396–405.
8. Goto H, Yang B, Petersen D, Pepper KA, Alfaro PA, Kohn DB, Reynolds CP. Transduction of green fluorescent protein increased oxidative stress and enhanced sensitivity to cytotoxic drugs in neuroblastoma cell lines. Mol Cancer Ther. 2003; 2:911–7.
9. Carr-Wilkinson J, Griffiths R, Elston R, Gamble LD, Goranov B, Redfern CP, Lunec J, Tweddle DA. Outcome of the p53-mediated DNA damage response in neuroblastoma is determined by morphological subtype and MYCN expression. Cell Cycle. 2011; 10:3778–87.
10. Keshelava N, Davicioni E, Wan Z, Ji L, Ji L, Sposto R, Triche TJ, Reynolds CP. Histone deacetylase 1 gene expression and sensitization of multidrug-resistant neuroblastoma cell lines to cytotoxic agents by depsipeptide. J Natl Cancer Inst. 2007; 99:1107–19.
11. Cohn SL, Salwen H, Quasney MW, Ikegaki N, Cowan JM, Herst CV, Kennett RH, Rosen ST, DiGiuseppe JA, Brodeur GM. Prolonged N-myc protein half-life in a neuroblastoma cell line lacking N-myc amplification. Oncogene. 1990; 5:1821–7.
12. Caren H, Kryh H, Nethander M, Sjoberg RM, Träger C, Nilsson S, Abrahamsson J, Kogner P, Martinsson T. High-risk neuroblastoma tumors with 11q-deletion display a poor prognostic, chromosome instability phenotype with later onset. Proc Natl Acad Sci U S A. 2010; 107:4323–8.
13. Goldschneider D, Horvilleur E, Plassa LF, Guillaud-Bataille M, Million K, Wittmer-Dupret E, Danglot G, de Thé H, Bénard J, May E, Douc-Rasy S. Expression of C-terminal deleted p53 isoforms in neuroblastoma. Nucleic Acids Res. 2006; 34:5603–12.

14. Van Maerken T, Rihani A, Dreidax D, De Clercq S, Yigit N, Marine JC, Westermann F, De Paepe A, Vandesompele J, Speleman F. Functional analysis of the p53 pathway in neuroblastoma cells using the small-molecule MDM2 antagonist nutlin-3. Mol Cancer Ther. 2011; 10:983–93.
15. Michels E, Hoebeeck J, De Preter K, Schramm A, Brichard B, De Paepe A, Eggert A, Laureys G, Vandesompele J, Speleman F. CADM1 is a strong neuroblastoma candidate gene that maps within a 3.72 Mb critical region of loss on 11q23. BMC Cancer. 2008; 8:173.

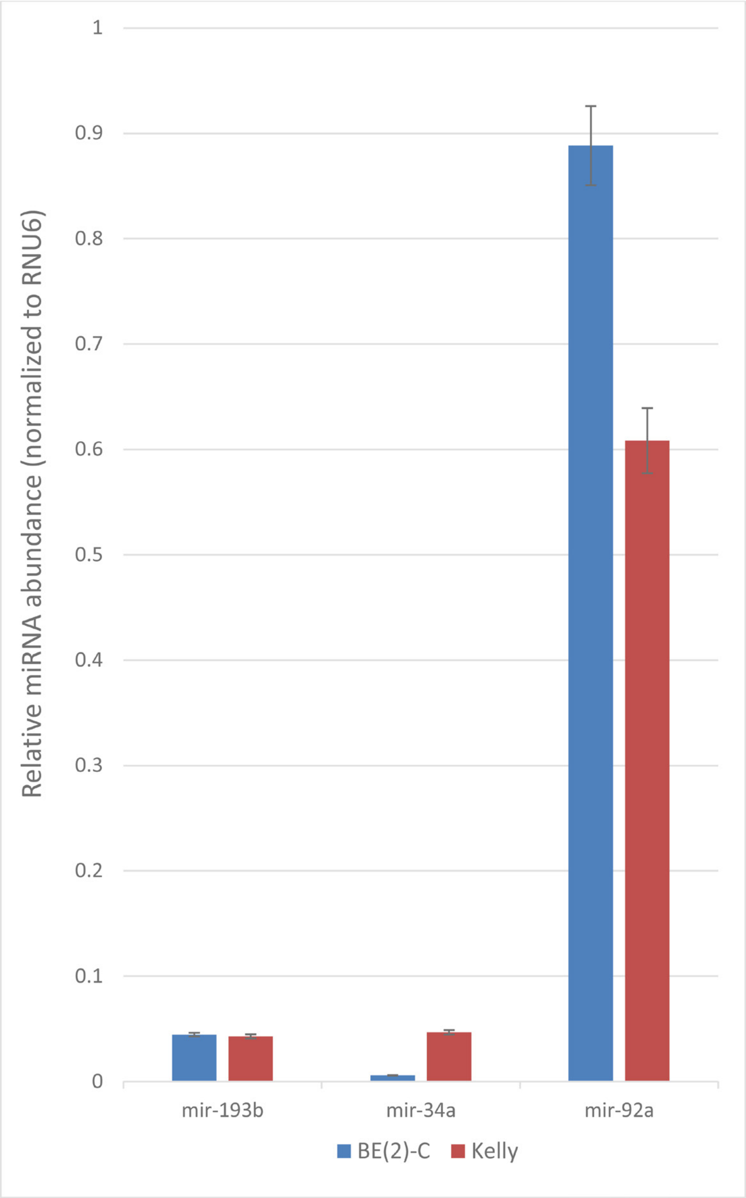

Supplementary Figure 1: Relative expression of selected miRNAs (mir-193b, mir-34a and mir-92a) in BE(2)-C and Kelly cells. RT-qPCR was performed on RNA isolated from BE(2)-C and Kelly according to description in Materials and Methods section. Raw fluorecence values (non-baseline corrected) were used to calculate mean PCR efficiencies in the LinRegPCR software [1]. PCR efficiency corrected Cq values were used to calculate the relative miRNA abundance according to 2^-Cq(MiR)/2^-Cq(RNU6). qPCR reactions were performed in triplicates on 3 independent biological replicates. Standard deviations were calculated taking into account the principle of error propagation (including technical and biological replicated and standard deviations from PCR efficiency determinations).

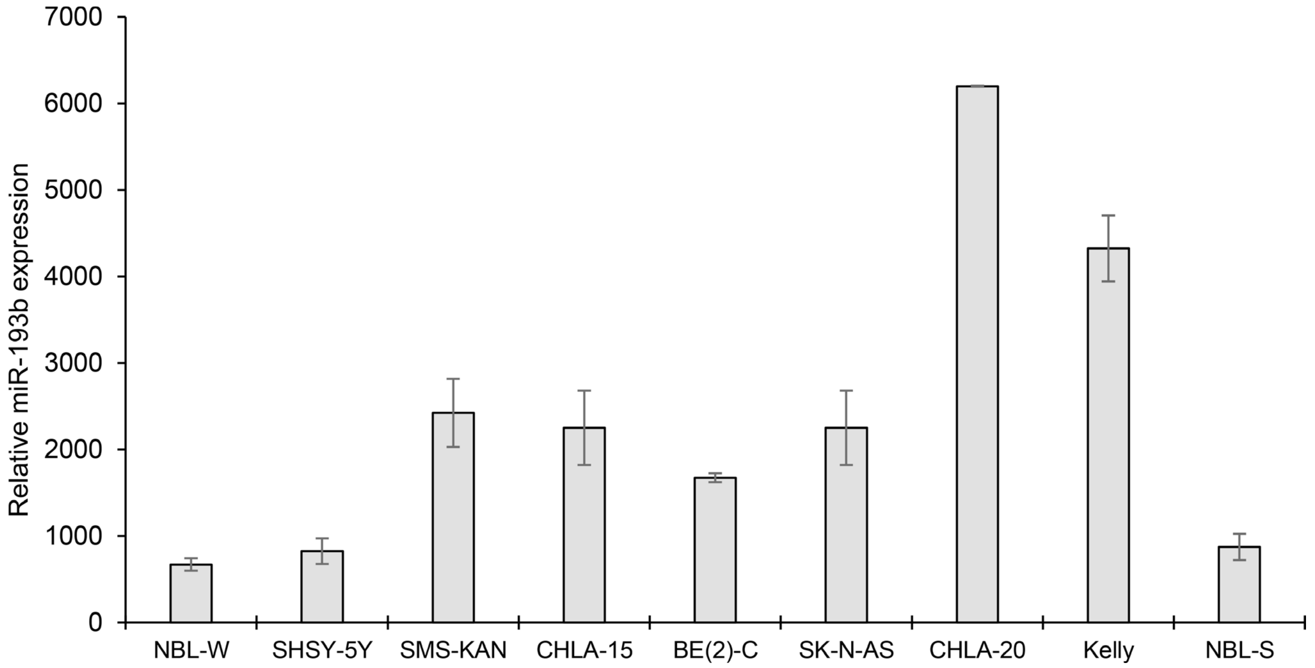

Supplementary Figure 2: MicroRNA-193b expression in neuroblastoma cell lines transfected with control or miR-193b mimics. 24 hours post-transfection, the total RNA was isolated, reverse transcribed into cDNA and the expression of miR-193b was analyzed by qRT-PCR. The expression levels of mature miR-193b were evaluated using the ΔΔCT comparative cycle threshold method. MiRNA expression levels of miR-193b mimic-transfected cells were calculated relative to miRNA expression levels of cells transfected with control mimics whose mean miRNA concentrations were set to 1. RNU6 was used as housekeeping genes. The experiment was performed at least two times giving similar results, and the result from one representative experiment is shown. Results are given as mean ± STDV from three independent samples and triplicate qPCR reactions.

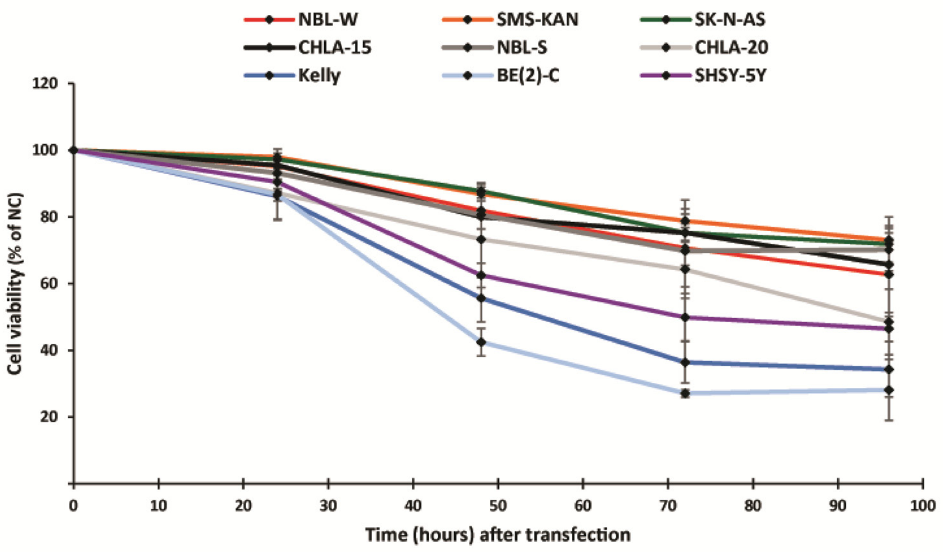

Supplementary Figure 3: miR-193b reduces cell viability in neuroblastoma cell lines. Data from Figure 2 including standard deviations.

```
Human             UAAUGAGAGGUGGCUUUUGCGGCCAGUAUUAGACUGG-AAGUUCAUACCUAAGUACUGUAAUAA
Chimp             UAAUGAGAGGUGGCUUUUGCGGCCAGUAUUAGACUGG-AAGUUCAUACCUAAGUACUGUAAUAA
Gorilla           UAAUGAGAGGUGGCUUUUGCGGCCAGUAUUAGACUGG-AAGUUCAUACCUAAGUACUGUAAUAA
Orangutan         UAAUGAGAGGUGGCUUUUGUGGCCAGUAUUAGACUGG-AAGUUCAUACCUAAGUACUGUAAUAA
Gibbon            UAAUGAGAGGUGGCUUUUGCGGCCAGUAUUAGACUGG-AAGUUCAUACCUAAGUACUGUAAUAA
Rhesus            UAAUGAGAGGUGGCUUUUGCGGCCAGUAUUAGACUGG-AAGUUCAUACCUAAGUACUGUAAUAA
Macaqee           UAAUGAGAGGUGGCUUUUGCGGCCAGUAUUAGACUGG-AAGUUCAUACCUAAGUACUGUAAUAA
Baboon            UAAUGAGAGGUGGCUUUUGCGGCCAGUAUUAGACUGG-AAGUUCAUACCUAAGUACUGUAAUAA
Green monkey      UAAUGAGAGGUGGCUUUUGCGGCCAGUAUUAGACUGG-AAGUUCAUACCUAAGUACUGUAAUAA
Marmoset          UAAUGAGAGGUGGCUUUUGCGGCCAGUAUUAGACUGG-AAGUUCAUACCUAAGUACUGUAAUAA
Squirrel monkey   UAAUGAGAGGUGGCUUUUGCGGCCAGUAUUAGACUGG-AAGUUCAUACCUAAGUACUGUAAUAA
Mouse             UAAUGAGAGGUGGCUCUUGCGGCCAGUAUUAGACUGG-AAGUUCACACCUAAGUACUGUAAGAA
Rat               UAAUGAGAGGUGGCUCUCG-GGCCAGUAUUAGACUGG-AAGUUCACACCUAAGUACUGUAAGAA
Rabbit            UAAUGCGCGGUGGCUUUCGCGGCCAGUGGUAGGCUGGCCCGUUCACACCUGAGUACUGUAAUAA
```

Supplementary Figure 4: The miR-193b binding site in the 3′-UTR of MYCN (highlighted in yellow) is highly conserved.

A

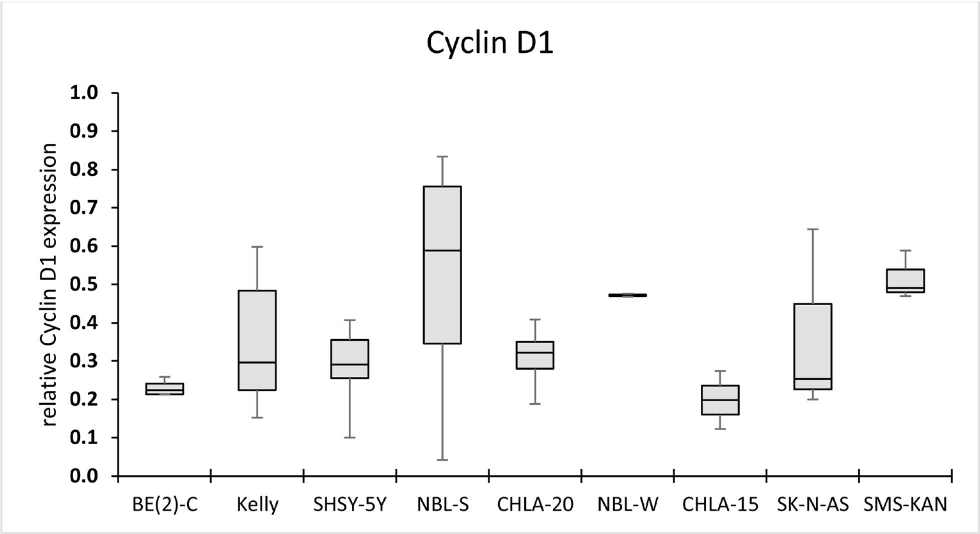
Cyclin D1
relative Cyclin D1 expression
1.0
0.9
0.8
0.7
0.6
0.5
0.4
0.3
0.2
0.1
0.0
BE(2)-C
Kelly
SHSY-5Y
NBL-S
CHLA-20
NBL-W
CHLA-15
SK-N-AS
SMS-KAN

B

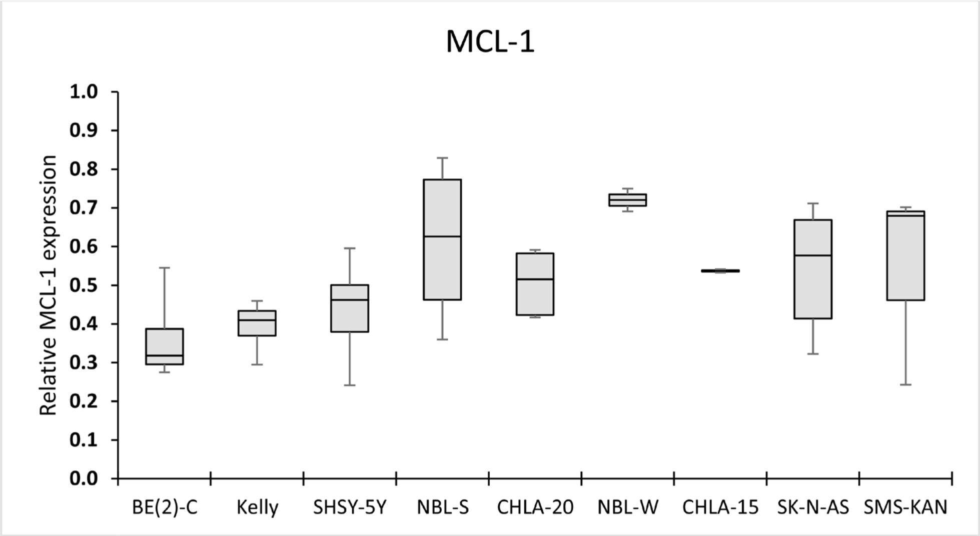
MCL-1
Relative MCL-1 expression
1.0
0.9
0.8
0.7
0.6
0.5
0.4
0.3
0.2
0.1
0.0
BE(2)-C
Kelly
SHSY-5Y
NBL-S
CHLA-20
NBL-W
CHLA-15
SK-N-AS
SMS-KAN

C

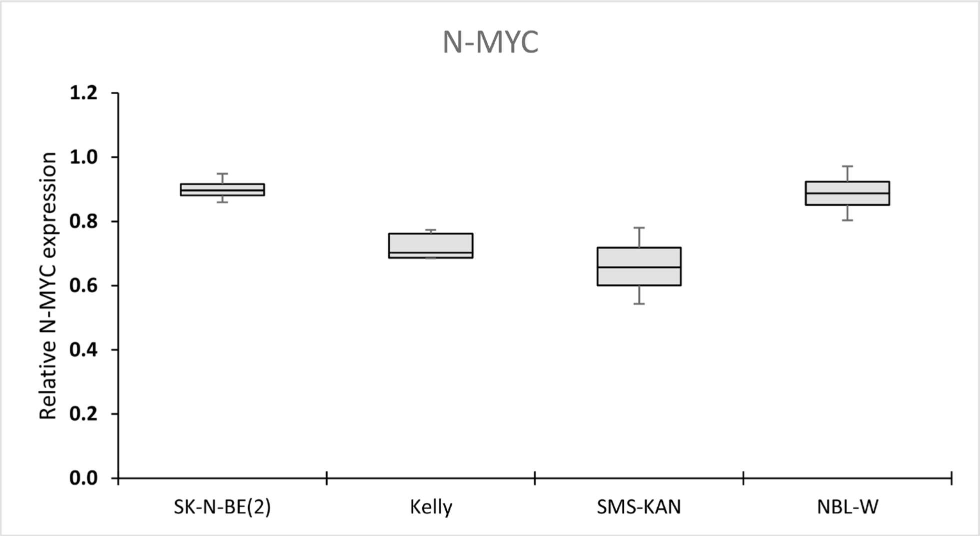

D

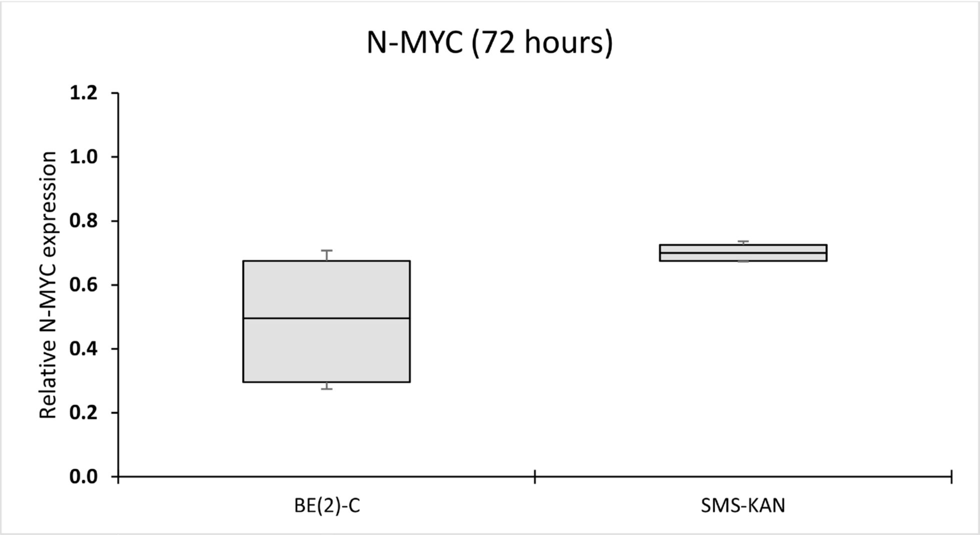

Supplementary Figure 5: MicroRNA-193b reduces the expression of cyclin D1 (**A**), MCL-1 (**B**) and N-MYC (**C** and **D**). Cells were transfected with miR-193b mimics or control mimics. 24 or 72 hours post-transfection, cells were harvested and protein expression was assessed by Western Blot. Blot intensities were quantified using ImageJ. The densitometric values were normalized to Actin. The boxplots (with median, 1st quantiles, 3rd quantile, maximum and minimum from at least two independent experiments) show protein expression of miR-193b-transfected cells relative to control-transfected cells (normalized values set to 1).

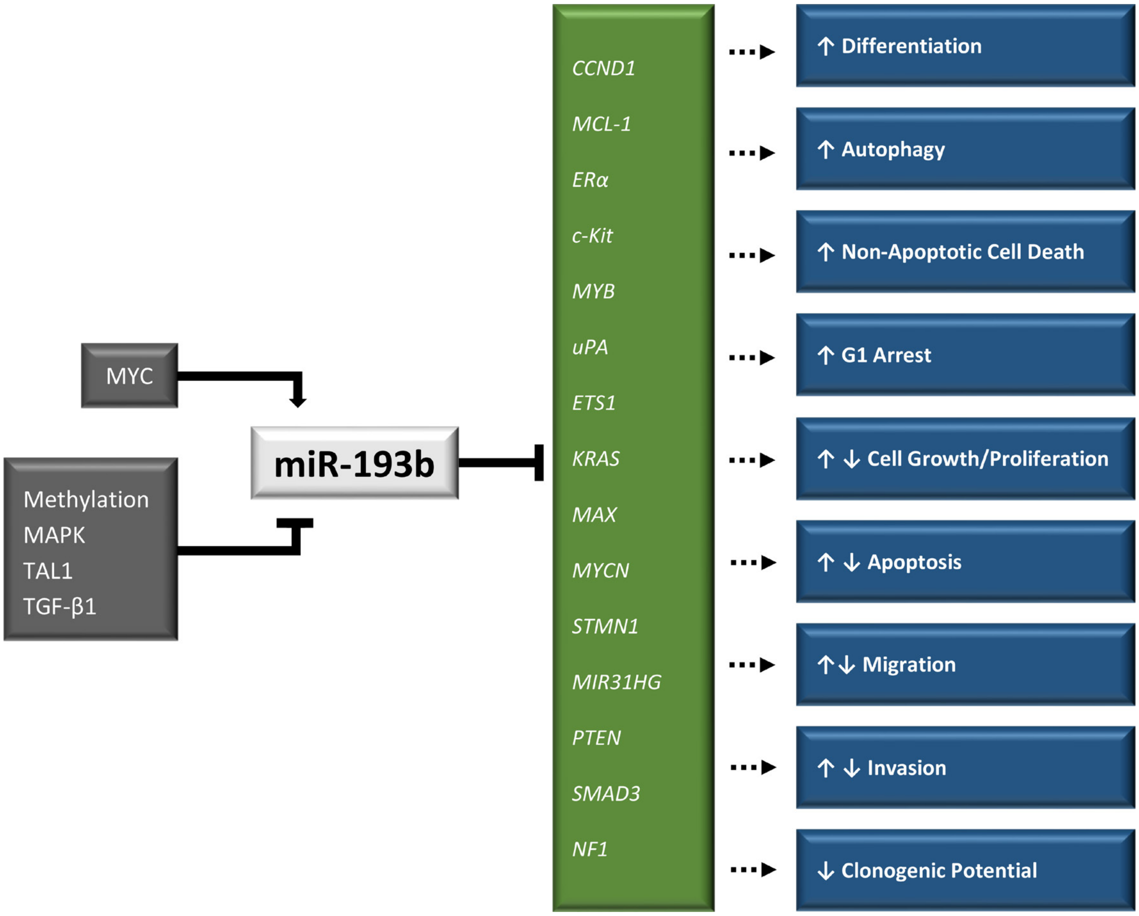

Supplementary Figure 6: Overview of miR-193b regulation and functions. MiR-193b expression is negatively regulated by gene methylation, MAPK and TGF-β1 signaling, and by TAL1, and positively regulated by MYC. MiR-193b affects multiple hallmarks of cancer by targeting numerous tumor suppressors and oncogenes.

REFERENCE

1. Ruijter JM, Ramakers C, Hoogaars WMH, Karlen Y, Bakker O, van den Hoff MJB, Moorman AFM. Amplification efficiency: linking baseline and bias in the analysis of quantitative PCR data. Nucleic Acids Res. 2009; 37:e45.
